# Phytochemical Analysis of the Methanolic Extract and Essential Oil from Leaves of Industrial Hemp *Futura 75* Cultivar: Isolation of a New Cannabinoid Derivative and Biological Profile Using Computational Approaches

**DOI:** 10.3390/plants11131671

**Published:** 2022-06-24

**Authors:** Simona De Vita, Claudia Finamore, Maria Giovanna Chini, Gabriella Saviano, Vincenzo De Felice, Simona De Marino, Gianluigi Lauro, Agostino Casapullo, Francesca Fantasma, Federico Trombetta, Giuseppe Bifulco, Maria Iorizzi

**Affiliations:** 1 Department of Pharmacy, University of Salerno, Via Giovanni Paolo II 132, 84084 Salerno, Italy; sdevita@unisa.it (S.D.V.); glauro@unisa.it (G.L.); casapullo@unisa.it (A.C.); 2 Department of Pharmacy, University of Naples, Via Domenico Montesano, 49, 80131 Naples, Italy; claudia.finamore@unina.it (C.F.); sidemari@unina.it (S.D.M.); 3 Department of Biosciences and Territory, University of Molise, Contrada Fonte Lappone, 86090 Isernia, Italy; mariagiovanna.chini@unimol.it (M.G.C.); saviano@unimol.it (G.S.); defelice@unimol.it (V.D.F.); fantasma@unimol.it (F.F.); 4 Societa Cooperativa Agricola MarcheSana, Localita San Biagio 40, 61032 Fano, Italy; federico.trombetta@gmail.com

**Keywords:** *Cannabis sativa* L. vr. *Futura 75*, cannabinoids, phytochemicals, essential oil, biological profile, inverse virtual screening

## Abstract

*Cannabis sativa* L. is a plant belonging to the Cannabaceae family, cultivated for its psychoactive cannabinoid (Δ^9^-THC) concentration or for its fiber and nutrient content in industrial use. Industrial hemp shows a low Δ^9^-THC level and is a valuable source of phytochemicals, mainly represented by cannabinoids, flavones, terpenes, and alkaloids, with health-promoting effects. In the present study, we investigated the phytochemical composition of leaves of the industrial hemp cultivar *Futura 75*, a monoecious cultivar commercially used for food preparations or cosmetic purposes. Leaves are generally discarded, and represent waste products. We analyzed the methanol extract of *Futura 75* leaves by HPLC and NMR spectroscopy and the essential oil by GC-MS. In addition, in order to compare the chemical constituents, we prepared the water infusion. One new cannabinoid derivative (**1**) and seven known components, namely, cannabidiol (**2**), cannabidiolic acid (**3**), β-cannabispirol (**4**), β-cannabispirol (**5**), canniprene (**6**), cannabiripsol (**7**), and cannflavin B (**8**) were identified. The content of CBD was highest in all preparations. In addition, we present the outcomes of a computational study focused on elucidating the role of 2α-hydroxy-Δ^3,7^-cannabitriol (**1**), CBD (**2**), and CBDA (**3**) in inflammation and thrombogenesis.

## 1. Introduction

*Cannabis sativa* L., commonly called “hemp”, belongs to the Cannabaceae family, is a well-known dioecious plant, and is among the most used and cultivated plants worldwide as it grows in variable habitats, soils, altitudes, and climate conditions [1]. The plant contains over 400 bioactive components such as cannabinoids, terpenes, flavonoids, and other phenolic compounds that have beneficial effects on the human body [2,3,4,5].

*C. sativa* L. can be differentiated into two distinct chemotypes based on the content of the principle psychoactive component, Δ^9^-tetrahydrocannabinol (Δ^9^-THC). The drug-type plant (marijuana or indica), which is rich in Δ^9^-THC, is used for medicinal or recreational purposes [6,7]; the second chemotype, the fiber-type *Cannabis*, commonly known as hemp (or industrial hemp), has a content of Δ^9^-THC below the legal limit of 0.2–0.3% [8,9], is rich in non-psychoactive cannabinoids, and is used for both textile or food purposes. Currently, 79 hemp varieties have been approved for commercial use by the European Community [10]. Recently Cannabis strains have been grouped into three types: Type I (high Δ^9^-THC content), Type II (various Δ^9^-THC to CBC ratios), and Type III (high CBD content) [11].

Hemp (fiber-type plants) is characterized by a complex chemical composition that includes terpenoids, sugars, alkaloids, stilbenoids, polyphenols, quinones, and cannabinoids, the specific compounds of this plant [12], which feature a characteristic C21 and C22 terpeno-phenolic backbone. Reports suggest that more than 100 cannabinoids are known; most of these are produced in their acid forms, such as cannabidiolic acid (CBDA), cannabigerolic acid (CBGA), and cannabichromenic acid (CBCA), then converted to their respective neutral counterparts, namely, cannabidiol (CBD), cannabigerol (CBG), and cannabichromene (CBC) [13].

*Cannabis sativa* L. has a wide range of therapeutic applications against several diseases, and many review articles have detailed its chemistry [14], the structure–activity relationship [15,16], and the complex biosynthesis of phytocannabinoids [6,17]. Major cannabinoids identified in *C. sativa* L. in addition to Δ^9^-THC include CBD, tetrahydrocannabivarin (THCV), cannabinol (CBN), cannabigerol (CBG), and cannabichromene (CBC) [14].

From a pharmaceutical point of view, phytocannabinoids are the most studied secondary metabolites on account of their ability to interact with the “endocannabinoid system” and/or with other kinds of pharmacological targets, including non-cannabinoid receptors. They have been found to possess a wide range of ubiquitous neuromodulatory actions and have a fundamental role in the physiology of most organisms [18]. Natural, endogenous, or synthetic cannabinoids may be used in cancer treatment, as they seem to induce autophagy and apoptosis, interfere with the cell cycle, and prevent angiogenesis [19,20]. Finally, an amelioration of the inflammatory state has been detected in several cases [21,22,23,24,25] and makes natural or synthetic cannabinoids interesting as multifaced molecular platforms.

CBD is an inactive psychotropic component and its levels tend to be higher in *C. sativa* cultivated for seed or fiber [26]. Many studies reported in the literature have proven that CBD has anxiolytic and neuroprotective properties [20,27], antioxidant [28], and anti-epileptic activity [6], while other derivatives, such as CBG, show antibacterial and anti-inflammatory properties [29]. Cannabinoids act as neuroprotective agents in Alzheimer’s and Parkinson’s diseases, exert anticancer activity [20] and exhibit palliative effects in cancer patients, preventing nausea and pain and stimulating appetite [30]. Prescriptions for medical cannabis are increasing in Italy, in line with many other countries where its therapeutic use is authorized thanks to its positive role in the treatment of various diseases. As a result, Italian galenic pharmacies are authorized to prepare precise doses of cannabis for vaping, herbal teas, resins, micronized capsules, and oils [31]. Recently, the U.S. Food and Drug Administration approved Epidiolex (CBD) oral solution for the treatment of seizures associated with two rare and severe forms of epilepsy, Lennox–Gastaut syndrome and Dravet syndrome [32]. CBD and its derivatives are the most therapeutically interesting compounds, despite being degradable and challenging to insert into a formulation capable of releasing a suitable therapeutic dose due to their chemical and lipophilic nature [21].

*Cannabis* is cultivated for its seeds for human or animal consumption [33]; hemp flour and oil are being developed as key ingredients for inclusion in a variety of foods [3] as a source of nutrients and non-nutrient compounds [34,35] with nutraceutical activity, as well as for application in the cosmetic industry. Hemp seeds have a high content of readily digestible proteins, lipids, and polyunsaturated fatty acids (PUFA) and a favorable omega-6 PUFA acid to omega-3 PUFA ratio [36]. *Cannabis* provides raw materials for many diverse uses, e.g., fiber in the textile and paper industry, oil production, as a construction material [37], in the automotive industry, in varnishes and inks [38], and as plant biomass for bioenergy production [39].

The interest in this plant has increased following the actual international trends toward use for therapeutic purposes, and monoecious varieties have been selected in modern times to reduce agronomic problems, although a small percentage of monoecious plants can naturally occur. In Italy, common modern monoecious varieties are *Futura 75* or *Felina 32*, while traditional varieties are dioecious, such as Finola or Carmagnola [40]. *Futura 75* is a cultivar of French origin; with a low Δ^9^-THC content (<0.2%), it is mainly utilized to produce seeds and fiber. Compared to other cultivars, *Futura 75* is a late crop, thus being more suitable for cultivation in mountainous regions [10,41]. Previous studies have reported antioxidant, anti-inflammatory, and antimycotic properties of the certified hemp variety “*Futura 75*” [42], whose essential oil from inflorescences showed antibacterial, anti-proliferative [43], and insecticidal effects [44]. Recently, these anti-inflammatory, anti-proliferative, and antimycotic properties have been detected in water extracts from inflorescences of *Futura 75* [45], and various common and emerging techniques have been reported to recover most phenolic and terpene compounds [46].

In the Abruzzo and Marche regions (central Italy), hemp cultivation is the object of renewed interest, and therefore the selection of *Futura 75* was made considering the local climatic and soil characteristics. When *Futura 75* variety is grown for seed and fiber production, the residual green parts, leaves, and stems are considered waste material.

The Italian MarcheSana Company (CANNAPA^®^) biologically grows the *Cannabis sativa* L. vr. *Futura 75*, and the inflorescences, leaves, and seeds are commercially used for food preparations such as herbal teas, oil, or cosmetic purposes.

Recently, considerable attention has been devoted to the recovery of waste products from agricultural and/or food processing industries, as biomass and byproducts are often sources of compounds with technological and nutritional properties.

As our current interest involves the chemistry of biologically active natural products (NPs), we investigated the chemical constituents of the residual green parts (leaves) from *C. sativa* L. vr. *Futura 75*, which are generally discarded, to support the valorization of locally produced plants and their use as pharmaceuticals or as health-promoting products. Prompted by the complex mechanisms involved in biological processes such as inflammation, here we present the outcomes of a computational study focused on elucidating the role of three secondary metabolites of *Futura 75*, namely, and a newly identified cannabitriol (**1**), CBD (**2**), and CBDA (**3**), in inflammation and thrombogenesis.

In this context, this study aimed to evaluate the phytochemical profile of methanol extracts, the essential oil composition (GC-MS), and the herbal infusion chemical components from leaves of *C. sativa* L. vr. *Futura 75*. A new CBD derivative (**1**) was isolated from methanolic extract, together with seven additional known compounds (**2**–**8**). The structural elucidation of all compounds was established based on 1D and 2D NMR experiments such as ^1^H- and ^13^C-NMR, ^1^H-^1^H-COSY, HSQC, HMBC, and ESIMS data, as well as comparisons with published data. We additionally present the outcomes of a computational study focused on elucidating the role of the newly identified cannabitriol (**1**), as well as CBD (**2**) and CBDA (**3**), in inflammation and thrombogenesis.

## 2. Results and Discussion

### 2.1. Phytochemical Analysis of Methanol Extracts

Methanolic crude extracts of *Cannabis* L. vr. *Futura 75* leaves were subject to extraction and chromatographic analyses. Four extracts were obtained using n-hexane, CHCl_3_, n-BuOH, and water following the modified Kupchan’s partitioning procedure [47].

**Figure 1:**
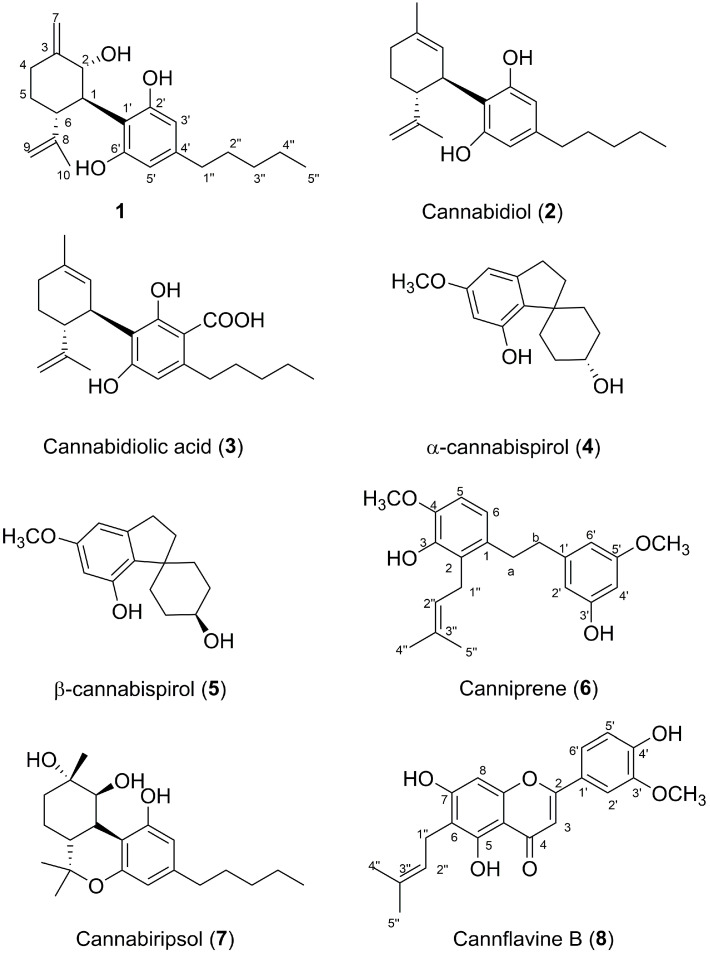
Natural compounds isolated from the leaves of *Cannabis sativa* L. vr. *Futura 75*.

Purification of the n-hexane and the CHCl_3_ fractions led to the isolation of one new cannabinoid component (**1**) along with seven known components **2**–**8** identified as cannabidiol (**2**) [48], cannabidiolic acid (**3**) [48], α-cannabispirol (**4**) [49], β-cannabispirol (**5**) [50], canniprene (**6**) [51], cannabiripsol (**7**) [52], and cannflavin B (**8**) [48] (Figure 1).

Structural elucidation was performed by comparing physical features, NMR spectroscopic data (1D and 2D experiments), and spectrometric analysis data (ESI-MS) with previously reported results (see Supplementary Materials, Figures S1–S12).

Compound **1** was obtained as an optically active white powder. The molecular formula was determined to be C_21_H_30_O_3_ from its positive-mode ESI-MS [M + H]^+^ peak at *m*/*z* 331. The structure of compound **1** was elucidated by extensive NMR correlation spectroscopy COSY, HSQC, and HMBC experiments (Figures S1–S5). The ^1^H and ^13^C-NMR spectroscopic data (CD_3_OD) indicated the presence of a cannabidiol-type skeleton [53] with a tetrasubstituted aromatic moiety and a linear pentyl chain (Table 1). ^1^H NMR spectrum showed signals due to a tertiary methyl group (δ_H_ 1.56, H_3_-10), one primary methyl group at δ_H_ 0.90 (3H, t, *J* = 7.0, H-5″), two aromatic proton signals partially overlapped at δ_H_ 6.11 and δ_H_ 6.09 (each s, H-3′ and H-5′ respectively), and two olefinic protons (δ_H_ 4.37 and 4.59, each brs, H_2_-9). In addition, ^1^H NMR revealed the presence of signals for two mutually coupled olefinic protons at δ_H_ 5.00 and 4.76 (each brs) and a broad doublet at δ_H_ 4.73, which is indicative of an oxygenated methine proton. ^1^H-^1^H-COSY implied connectivities in the cyclohexane ring from the H-2 methine proton to H_2_-4 through H-1, H-6, and H_2_-5.

The HSQC experiment allowed us to associate all proton signals with those of directly linked carbons. A comparative analysis of 2D experiments of **1** with those of CBD (**2**) revealed that the main differences were observed in the substitution pattern of the cyclohexane ring, with the presence of signals for an exomethylene functionality C-3/C-7 (δ_H_ 5.00 and 4.76, each brs) instead of a double bond between C-2 and C-3. This hypothesis was supported by the HMBC correlations between H_2_-7 (δ_H_ 5.00 and 4.76) and C-3 (δ_C_ 153.1), C-2 (δ_C_ 73.9), and C-4 (δ_C_ 35.4).

The location of an extra hydroxyl group at C-2 (δ_H_ 4.73, δ_C_ 73.9) was established by COSY correlation between H-2 (δ_H_ 4.73) and H-1 (δ_H_ 3.15) and was confirmed by HMBC, which showed correlations H-2/C-6 (δ_C_ 48.2) and C-7 (δ_C_ 104.7) (see Section 3). The 2α-hydroxy orientation at C-2 was suggested on the basis of the large H-2 vicinal coupling constant (*^3^J*_H-2, H-1_) (br d, *J* = 11.0 Hz,), indicating the *trans*-diaxial orientation of H-2 and H-1. The *trans* absolute configuration *1R*, *6R* (*^3^J*_H-1, H-6_ = 11.0 Hz) was assumed to be the same as that of CBD [48,53].

The same substitution pattern in the cyclohexane ring was previously found in Δ^9,11^-hexahydrocannabinol [54], and these results are in line with data recently reported by Chianese et al. [55]. Based on these experiments, the chemical structure of **1** was elucidated as *1R*, *2R*, *6R* 2α-hydroxy-Δ^3,7^-cannabitriol.

### 2.2. Analysis of Volatile Compounds from Leaves of C. sativa L. vr. Futura 75

The essential oil (EO) was prepared via hydrodistillation, and was generally obtained as the primary product from female flowers. We prepared the EO using dried residual green parts (leaves). The phytochemical composition of EO is shown in Table 2 and the GC-MS profile (TIC) is presented in Figure S13.

Eighty-two compounds were identified, corresponding to 97.45% of the total area of the extract composition. Sesquiterpenes represented the most abundant class (32.71%), followed by oxygenated sesquiterpenes (28.04%), cannabinoids (32.11%), and monoterpenes (1.28%). Major sesquiterpenes included β-caryophillene (13.82%), caryophillene oxide (5.70%), α-humulene (5.33%), bisabolol oxide (5.12%), aromadendrene epoxide (4.41%), caryophillene-14-hydroxy (3.44%), and γ-himachalene (3.5%). Among cannabinoids, CBD (**2**) was the dominant constituent (28.5%). Cannabicitran (1.6%), cannabichromene (0.6%), cannabidivarin (0.7%), cannabicyclol (0.26%), cannabinol (0.14%), cannabigerol (0.1%), and Δ^9^-THC in trace amounts (0.15%) were detected as well. Monoterpenes and oxygenated monoterpenes were poorly present in the essential oil of *Futura 75* leaves, with α-pinene (0.58%) and limonene (0.19 %) as the most abundant compounds among hydrocarbons. The loss of terpenes and the oxidative reaction of sesquiterpenes is induced by the drying process, which according to Fiorini [56] leads to the formation of caryophyllene oxide and other oxidized derivatives. The Δ^9^-THC content of the analyzed leaves was found to be below the limits imposed by Italian law (THC < 0.2%). Table 2 reports the chemical composition of the EO and the experimental retention indices as compared with the retention indices reported in the literature [57,58,59].

Compared to previous studies, two major differences can be noted in our analysis. First, our EO showed a significantly high CBD content, probably due to the drying process of the fresh leaves. Of further interest is the presence of β-caryophyllene, a component with remarkable biological properties, in higher concentrations than previously identified. The sesquiterpene profile of *Futura 75* EO is in agreement with that previously found in the essential oil of *C. sativa* L. [60] and in *Futura 75* (aerial parts) [43,44,61], in which β-caryophyllene, caryophyllene oxide, and α-humulene were the most representative terpenoids. From a pharmaceutical point of view, β-caryophyllene, although a sesquiterpene, can selectively bind to type 2 cannabinoid receptors (CB2), where it shows significant anti-inflammatory activity without any psychotomimetic effects. Furthermore, it has been proven to exert gastric cytoprotective activity as an anti-inflammatory agent and may ameliorate the symptoms of anxiety and depressive disorders in a rat model [20,62]. Therefore, thanks to its neuroprotective activities combined with CBD content, this EO could be a potential candidate against neurodegenerative diseases.

### 2.3. Major Phytochemical Components in Futura 75 Water Infusion

Medical cannabis is legally available in several countries and has significant variations in phytocannabinoids content according to the cultivar and geographical area. Patients consume medical cannabis in its dried form and in a variety of ways, including vaping, food preparation, or as infusions, herbal teas, decoctions, or infused edible oils. The main objective of the present study was to compare the chemical profile of methanol extract with the components detected after water infusion of *Futura 75* dried leaves. The water infusion (WI) was submitted to the modified Kupchan’s partitioning procedure and four extracts were obtained (n-hexane, CHCl_3_, n-BuOH, and water extract). Few components could be detected in the WI n-hexane extract, which resulted in a mixture of fatty acids and residual amounts of cannabinoid derivatives. Much more interestingly was the WI chloroform extract, which showed a high content of cannabinoid derivatives in which CBD (**2**) was predominant, with traces of CBDA (**3**) (see Section 3.3 and Figures S6 and S7). In ^1^H NMR spectrum analysis, the n-BuOH extract was found to be rich in monosaccharide and polysaccharide components (3.0–5.0 ppm) and showed a complex mixture of polyphenols (6.0–8.5 ppm). Finally, the main detectable components in the aqueous WI extract were mono- and polysaccharides (3.0–5.0 ppm), with small amounts of free amino acids (0.9–3.2 ppm) and organic acids (1.7–3.2 ppm). In summary, the infusion of *Futura 75* leaves showed a complex metabolite composition largely dominated by primary plant metabolites such as carbohydrates and especially by CBD and its derivatives, which are secondary metabolites with nutraceutical and pharmaceutical value. Therefore, comprehensive chemical analyses such as those presented in the current study can help to facilitate the adoption by the medical community of products based on medical cannabis extracts.

### 2.4. Inverse Virtual Screening

Inverse Virtual Screening (IVS) is a computational technique that aims to highlight the most promising protein partner(s) for a molecule among a large set of possible targets [63,64,65]. In detail, IVS is structured into three steps: (1) molecular docking of the studied compound(s) against the target panel; (2) normalization of each ligand/target complex binding affinity; and (3) analysis of the obtained results. This approach is particularly useful in the study of NPs because they are usually extracted and purified in small amounts and because it avoids extensive biological studies. Therefore, narrowing down the list of possible interactors can result in a more efficient experimental procedure.

In the present study, **1**, **2**, and **3** were tested in silico against a panel of proteins involved in the acute inflammatory response (GO ID: 0002526, 3789 entries) that was previously prepared for the calculations using an automated workflow [66] (see Section 3.8 for details). After docking the three compounds against the whole panel and collecting the corresponding binding affinities, the results were normalized using a set of ten decoys that resemble the molecules of interest in terms of chemical properties while having different structures. The average binding affinity of these decoys on each target was used to generate the parameter V (see Section 3.9). This mathematical manipulation is helpful in identifying false-positive results derived from non-specific binding. The normalized results were filtered to keep only targets with calculated binding energy below −7.5 kcal/mol and a V value above 0.75.

The results obtained were then analyzed in order to highlight two key aspects: the top score (in terms of V or pure binding affinity) for each molecule (Table 3), and the most retrieved targets shared between the three compounds (Table 4).

The analysis of these results highlights two important factors: the three NPs showed their best interaction energies towards the same target (TNFα), and the top-retrieved proteins (i.e., the macromolecules that occurred the most in the filtered results) in common were prothrombin/thrombin (THR) and peroxisome proliferator-activated receptor gamma (PPARγ). This uniformity in the output data can be explained by the high structural similarity of **1**, **2**, and **3** (Figure 1). In detail, the 5-pentyl-1,3-benzyldiol moiety (also known as olivetol) is the common denominator in the three molecules; it provides two hydrogen bond acceptors/donors and a phenyl ring that can interact with aromatic residues, while the other 6-C ring and the pentyl chain are instead responsible for the hydrophobic contacts that sometimes represent the driving force of protein-ligand binding.

#### 2.4.1. TNFα

Interestingly, all three secondary metabolites show their best binding affinities towards a common target, tumor necrosis factor-alpha (TNFα). TNFα is a trimeric pro-inflammatory cytokine that activates the NF-κB and mitogen-activated protein kinase (MAPK)/AP-1 pathways [73,74].

Its complex signalling pathway and significance in several pathologies have been extensively reported [75,76], and only five direct inhibitors have been approved by the FDA thus far [77,78]. The development strategy of direct TNFα inhibitors is based on disrupting the symmetric structure of the trimer and avoiding its complete interaction with TNF receptors (TNFR). With this aim, the drug design process is focused on identifying suitable scaffolds with good pharmacological properties that can occupy the pocket between two contiguous protein chains [67,79]. Recently, Ma et al. [80] used a network analysis approach to identify potential targets and explain the anti-inflammatory effect of **2**, however, TNFα is not listed among the proposed interactors, despite the connection between cannabinoids and TNFα inhibition having been studied for a long time [81,82].

According to previous molecular docking studies [83,84] and based on the co-crystallized ligands, the key involved in binding with inhibitors is Tyr119; in addition to this, Leu57, Tyr59, Ser60, Leu120, Gly121, Gly122, and Tyr151 represent other important interaction sites [85].

**Figure 2:**
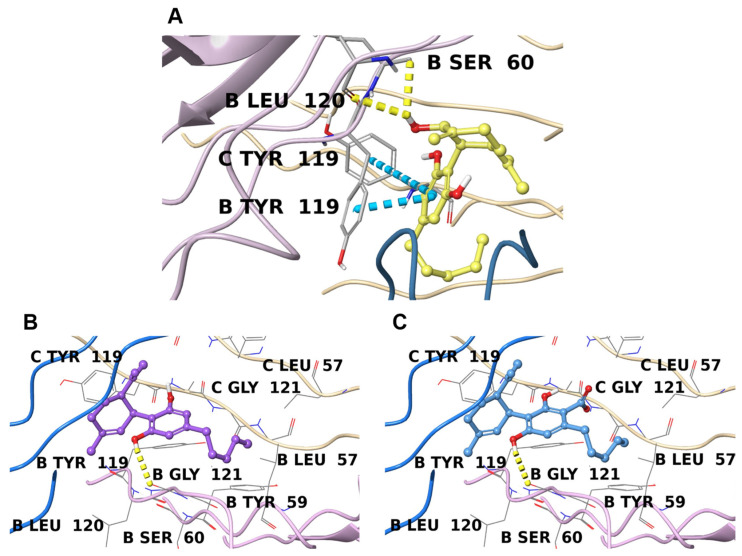
Binding poses of **1** (**A**), **2** (**B**), and **3** (**C**) inside the pocket of TNFα (PDB 6X83 for **1** and PDB 7KPA for **2** and **3**), with different ribbon colors for each monomer The hydrogen bonds are depicted as yellow dotted lines and π-π stacking interactions as cyan dotted lines. Interacting residues and other important binding site amino acids are labeled (chain name + residue).

Both **2** and **3** interact with the same crystallographic structure (PDB: 7KPA), and due to their lipophilic chemical structure, they are well inserted in the highly hydrophobic binding pocket (Figure 2B,C). Moreover, they form a hydrogen bond with Gly121, and their orientation in the cavity is almost identical. Moreover, they share a high degree of similarity with the binding mode shown by the co-crystallized TNFα inhibitor UCB-8733 (Figure S14B). Compound **1**, on the other hand, interacts better with another TNFα crystal structure (PDB: 6X83), and more interestingly, with an allosteric site that was only recently discovered (Figure 2A) [67]. Compound **1** appears to be perfectly inserted in this cavity, where it interacts at the interface between monomer B and C, leading to important π-π stacking with Tyr119 residues on both sides and forming two hydrogen bonds with Ser60 and Leu120 on chain B. In this way, TNFα is stabilized in this inactive state. This molecular orientation perfectly covers the two co-crystallized fragments in the original structure (Figure S14A) and corroborates the hypothesis that **1** can act as a disruptor of TNFα symmetry.

#### 2.4.2. Prothrombin/Thrombin

Prothrombin (coagulation factor II) is the inactive precursor form of thrombin (coagulation factor IIa), and is proteolytically cleaved into thrombin by factor Xa and factor Va [86]. In this way, the serine–protease enzymatic activity of thrombin (THR) is activated, converting fibrinogen into fibrin and initiating the clot formation process. Only a few direct inhibitors have been approved thus far because of the low “druggability” of THR [69,87], however, several studies have indicated that plant-derived secondary metabolites can perform a direct inhibitory activity [88,89,90,91]. Here, the UniProt ID indicates both the precursor and the mature form of the enzyme; therefore, it is simply addressed here as “THR”. THR is reported to have a catalytic binding site characterized by the triad His57, Asp102, and Ser195 [69,70] along with two positively charged exosites; exosite I is the destination for interaction with fibrinogen and is formed by the Arg76-Ile82 segment, whereas exosite II is larger and is responsible for the interaction with heparin, as delimited by Tyr89-Arg101, Arg126-Leu130, Glu164-Lys169, and Phe232-Phe245 [88,89,90,91,92].

The results we collected indicate P00734 as the most retrieved UniProt ID for the three NPs, with a calculated binding affinity below −8.0 kcal/mol, which provides a preliminary indication of possible interactions with this enzyme.

**Figure 3:**
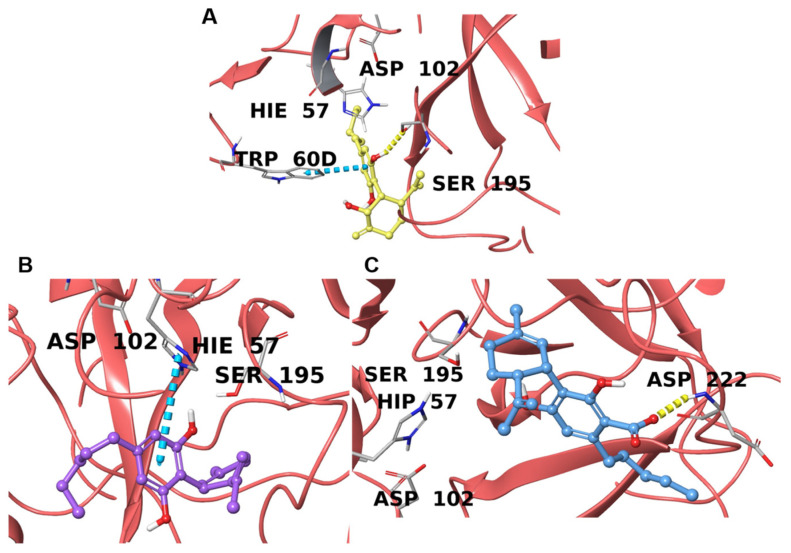
Binding poses of **1** (**A**), **2** (**B**), and **3** (**C**) in the catalytic domain of thrombin (PDBs 6ZUX, 6ZV8, and 1RD3, respectively) are represented with red ribbons. The hydrogen bonds are depicted as yellow dotted lines and π-π stacking interactions as cyan dotted lines. Interacting residues and catalytic triad amino acids are labeled.

From our analysis of the data, **1** and **2** interact directly with residues forming the catalytic triad; in detail, **1** forms a hydrogen bond with Ser195, while the aromatic moiety of **2** forms a π-π contact with His57, potentially hampering the proteolytic activity of THR (Figure 3A,B). Compound **3**, on the other hand, shows no relevant interactions with the triad; although the carboxyl oxygen forms a hydrogen bond with Asp222 on the external part of the exosite II, this apparently has no role in the inhibition of the enzyme (Figure 3C). For 6ZUX and 6ZV8, a co-crystallized compound was available, and the superimposition highlighted a similar spatial disposition of **2** and QQT (Figure S15B), whereas **1** and QQE showed only a partial overlap in the binding cavity (Figure S15A) despite interacting with the same amino acids. The hypothetical direct inhibition performed on THR by these compounds could explain the controversial cardiovascular effects, particularly related to coagulation, ascribed to cannabinoids [93]; however, future evaluations must be performed.

#### 2.4.3. PPARγ

The third putative target highlighted by the Inverse Virtual Screening method is the peroxisome proliferator-activated receptor γ (PPARγ). These nuclear factors form a heterodimer with retinoid X receptor (RXR); this complex translocates into the nucleus and recognizes PPAR response elements (PPREs) that encode for genes involved in metabolic, autoimmune, anti-cancer, and inflammatory pathways [71,94,95]. This makes them an interesting pharmacological target, especially for anti-diabetic drugs [94,96,97]. Based on data reported in previous studies, the binding site has a forked Y-shaped conformation containing four different sites, called P1-4 [98], that can accommodate different molecular platforms. This conformation is located deep in the space between helix 3 and helix 6 [98,99]. The binding site is formed by Asp166, Tyr192, Gln193, Tyr189, Leu196, Ala197, Lys201, Arg202, Glu203, Leu237, Val248, Tyr250, Asn335, Lys336, Asp337, Phe347, Glu351, and Tyr473 [71,100,101,102]. In addition, the co-crystallized ligand of 4PRG (GW0072, 072 in the crystal structure) showed a particular binding mode that involved Phe264, Arg280, Arg288, Tyr327, and Ser342.

**Figure 4:**
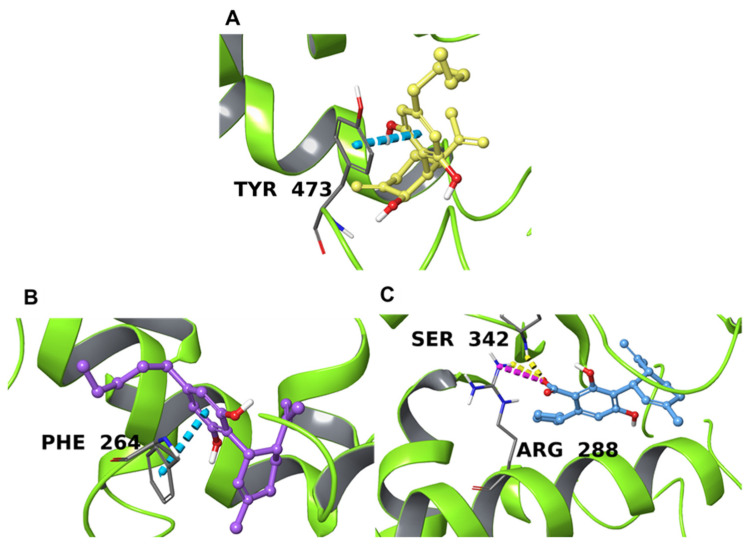
Binding poses of **1** (**A**), **2** (**B**), and **3** (**C**) in the catalytic domain of PPARγ (PDB 2ZK6 for **1** and 4PRG for **2** and **3**) depicted with lime green ribbons. The hydrogen bonds are depicted as yellow dotted lines, the salt bridges with magenta dotted lines, and π-π stacking interactions as cyan dotted lines. Interacting residues are labeled.

The action of endocannabinoids on PPARγ has already been described [103,104,105] as due to the presence of arachidonic acid derivatives in the endocannabinoid system [106], and Iannotti et al. have recently reported the action of cannabimovone on this nuclear factor [107], corroborating the hypothesis that other secondary metabolites of *C. sativa* could inhibit its action. The structure-activity relationship of known PPARγ ligands has demonstrated that full agonists, unlike partial agonists, interact with Tyr473 [71]. From our analysis of the resulting binding poses, **3** shows slightly better binding towards the protein target interacting with Arg288 and Ser 342 through the carboxyl portion of the molecule, while **2** forms a π-π interaction between its aromatic ring and Phe264 side chain (Figure 4B,C). The superposition of **2** and **3** with the co-crystallized ligand (GW0072, 072 in the crystal structure) highlighted that these two NPs occupy approximately the same volume of GW0072 and overlap with it, despite GW0072 being considerably larger than **2** and **3** (Figure S16B and S16C). Therefore, the good binding affinity calculated for **2** and **3** is mainly derived from hydrophobic contacts, while the small difference between the two compounds could be ascribed to the hydrogen bond and ionic interaction that involved **3**. Compound **1**, on the other hand, is located in the outer part of the binding domain (Figure 4C) and interacts with the side chain of Tyr473 through π-π stacking contact. Despite favorable contact with one of the key binding site residues, **1** did not overlap at all with the co-crystallized ligand (Figure S16A) due to the different nature of the ligand (C8-BODIPY, C08 in the crystal), which is a fatty acid analog able to bind PPARγ in the deep part of the pocket.

## 3. Materials and Methods

### 3.1. General Experimental Procedure

Specific rotations were measured on a Perkin Elmer 243 B polarimeter. An LTQ-XL mass spectrometer (Thermo Fisher Scientific, Waltham, MA, USA) was used to perform ESI-MS spectra.

NMR spectra were recorded on Varian Inova 400 and 500 spectrometers (^1^H at 400 MHz and ^13^C at 100 MHz, and ^1^H at 500 MHz and ^13^C at 125 MHz, respectively) equipped with SUN microsystem ultra 5 hardware. Coupling constants (*J* values) are reported in Hertz (Hz) and chemical shifts (δ) in ppm, referred to as CD_3_OD (δ_H_ 3.31 and δ_C_ 49.0) and pyridine-d_5_ (δ_H_ 8.74, 7.58, 7.22; δ_C_ 150.3, 135.9, 123.8). Spin multiplicities are provided as s (singlet), brs (broad singlet), d (doublet), t (triplet), or m (multiplet). ^13^C-^1^H connectivities were determined using HSQC and HMBC experiments, while ^1^H-^1^H connectivities were determined through COSY experiments. DCCC was performed using a DCC-A (Rakakikai Co., Tokyo, Japan) equipped with 250 columns (internal diameter 3 mm). Silica gel (200-400 mesh) was purchased from the Macherey-Nagel Company.

HPLC was performed using a Waters Model 510 pump equipped with a Waters Rheodine injector and a differential refractometer, model 401. The HPLC columns used were a Luna C18 (5 μm, 250 mm × 4.6 mm i.d.) (Phenomenex, Torrance, CA, USA) and Nucleodur C18 100-5 (10 μm, 250mm × 4.6 mm i.d.) (Macherey-Nagel, GmbH & Co., KG, Neumann-Neander-Str.6-8, Düren, Germany).

### 3.2. Plant Material

Fiber hemp plants (*C. sativa*) belonged to the monoecious *Futura 75* genotype (Plant Variety Catalogues, Databases), (batch number seeds: F1545—R154901B 01/2016). The dried leaves of *Cannabis Sativa* vr. *Futura 75* were kindly provided by the Italian agricultural cooperative society, MarcheSana (CANNAPA^®^) (Via di Villa Giulia, Loc. S. Biagio, Fano, Pesaro Urbino, Italy), and were certified as having a content of Δ^9^-THC below 0.2% (*w*/*w*).

The samples considered in this study were hemp varieties approved for commercial use by the European Union (https://eur-lex.europa.eu/legal-content/EN/TXT/?uri=CELEX%3A02013R1307-20220101 accessed on 1 June 2022), and leaves or inflorescences were commercialized for food purposes as flours, infusions, biscuits. Hemp inflorescences were manually separated from seeds, then the samples were stored at +4.0 °C until required for chemical analysis. A voucher specimen was deposited under No. CAN-31-2018 in the Herbarium of the University of Molise (Pesche, Isernia, Italy).

### 3.3. Extraction and Isolation

Hemp leaves (190 g) were ground in a mortar and extracted with methanol (3 × 1 L) at room temperature. The combined extracts (17.0 g) were concentrated and subjected to modified Kupchan’s partitioning procedure [47] as follows. The MeOH extract was dissolved in 10% aqueous methanol and partitioned against n-hexane, yielding 2.76 g of the n-hexane extract. The water content (% *v*/*v*) of the MeOH extract was adjusted to 40% and partitioned against CHCl_3_ to furnish a CHCl_3_ extract (5.65 g). The aqueous phase was concentrated to remove MeOH and then extracted with n-BuOH, yielding 3.74 g of glassy material (n-BuOH extract).

The CHCl_3_ extract (5.65 g) was submitted to droplet counter-current chromatography (DCCC) with CHCl_3_/MeOH/H_2_O (7:13:8) in the ascending mode (the lower phase was the stationary phase) at a flow rate of 8 mL/min, and 4 mL fractions were collected. The obtained fractions were monitored by TLC on Silica gel plates with CHCl_3_/MeOH/H_2_O (80:18:2) as eluent.

DCCC purification on 5.65 g of CHCl_3_ extract afforded seventeen fractions, several of which were purified by HPLC on a Luna C18 (5 μm, 250 mm × 4.6 mm i.d) using MeOH/H_2_O (7:3) + 0.1% TFA (trifluoroacetic acid) as eluent (flow rate 1.0 mL/min). Purification of Fraction 8 (13.8 mg) furnished 1.8 mg of pure α-cannabispiranol (**4**) (t_R_ = 7.82 min) and cannabiripsol (**7**) (t_R_ = 22.11 min). Fraction 10, in the same conditions, afforded 1.6 mg of pure β-cannabispiranol (**5**) (t_R_ = 11.2 min). Fraction 11 (51.2 mg) provided 4.2 mg of the new cannabitriol (**1**) (t_R_ = 20.3 min) and 5.3 mg of cannflavin B (**8**) (t_R_ = 30.0 min). Fraction 13 (53.8 mg) mainly contained canniprene (**6**) (5.6 mg; t_R_ = 8.4 min) and CBD (**2**) (9.2 mg; t_R_ = 22.8 min). Fractions 15 (97 mg) and 16 (193.2 mg) were purified using a semi-preparative Nucleodur 100-5 C18 column (10 μm, 250 mm × 4.6 mm i.d) (flow rate 4.0 mL/min) with MeOH/H_2_O (8:2) as eluent to obtain pure CBD (**2**) (89.3 mg; t_R_ = 22.9 min) and cannabidiolic acid (**3**) (74.3 mg; t_R_ = 28.4 min).

#### 3.3.1. 2α-Hydroxy-Δ^3,7^-Cannabitriol (**1**)

White powder; [α]_D_^25^ + 16.4 (c 0.12, MeOH); ^1^H NMR data (400 MHz) and ^13^C NMR data (100 MHz) in CD_3_OD are reported in Table 1; ESIMS (positive-ion mode) *m*/*z* 331 [M + H]^+^.

#### 3.3.2. Cannabidiol (**2**)

White powder; [α]_D_^25^-81.4 (c 0.60, MeOH); ^1^H NMR data identical to those previously reported in the literature [48]; ESIMS (positive-ion mode) *m*/*z* 315 [M + H]^+^.

#### 3.3.3. Cannabidiolic Acid (**3**)

White powder; [α]_D_^25^ -54.0 (c 0.12, MeOH); ^1^H NMR data identical to those previously reported in the literature [48]; ESIMS (positive-ion mode) *m*/*z* 359 [M + H]^+^.

#### 3.3.4. α-Cannabispiranol (**4**) and β-Cannabispiranol (**5**)

White powders; the ^1^H NMR data (C_5_D_5_N) were in agreement with the literature and led us to assign the stereochemistry at C-4 [49]; ESIMS (positive-ion mode) *m*/*z* 249 [M + H]^+^.

#### 3.3.5. Canniprene (**6**)

White powder; ^1^H NMR data (500 MHz, CD_3_OD): δ_H_ 1.66 (3H, s, H_3_-4″), 1.75 (3H, s, H_3_-5″), 2.67 (2H, m, H_2_-b), 2.74 (2H, m, H_2_-a), 3.34 (2H, d, *J* = 6.6 Hz, H_2_-1″), 3.70 (s, OCH_3_-7), 3.82 (s, OCH_3_-7′), 5.08 (1H, t, *J* = 6.6 Hz, H-2″), 6.18 (1H, s, H-4′), 6.20 (1H, s, H-2′), 6.22 (1H, s, H-6′), 6.59 (1H, d, *J* = 8.3 Hz, H-6), 6.70 (1H, d, *J* = 8.3 Hz, H-5); ^13^C NMR data (125 MHz, CD_3_OD): δ_C_ 18.1, 25.9, 26.1, 35.7, 39.3, 55.5, 56.5, 99.9, 106.5, 109.0, 109.8, 120.8, 125.1, 127.6, 131.3, 134.4, 145.2, 145.8, 146.9, 159.4, 162.2. ESIMS (positive-ion mode) *m*/*z* 343 [M + H]^+^.

#### 3.3.6. Cannabiripsol (**7**)

White powder; [α]_D_^25^ -73.0 (c 0.12, MeOH); the ^1^H NMR data were in agreement with the literature; ESIMS (positive-ion mode) *m*/*z* 349 [M + H]^+^.

#### 3.3.7. Cannflavin B (**8**)

White powder; ^1^H NMR data (400 MHz, CD_3_OD): δ_H_ 1.67 (3H, s, H_3_-4″), 1.79 (3H, s, H_3_-5″), 3.34 (2H, ovl, H_2_-1″), 3.96 (3H, s, OCH_3_), 5.24 (1H, t, *J* = 7.20 Hz, H-2″), 6.49 (1H, s, H-8), 6.63 (1H, s, H-3), 6.94 (1H, d, *J* = 8.0 Hz, H-5′), 7.47 (1H, ovl, H-2′), 7.50 (1H, d, *J* = 8.2 Hz, H-6′); ^13^C NMR data (100 MHz, CD_3_OD): δ_C_ 17.9, 22.2, 26.0, 56.6, 94.1, 104.0, 105.1, 110.4, 113.1, 116.6, 121.6, 123.4, 123.7, 132.0, 149.5, 151.9, 157.2, 160.0, 163.7, 165.8, 183.9. ESIMS (positive-ion mode) *m*/*z* 369 [M + H]^+^.

### 3.4. Essential Oil Preparation

20 g of dried leaves of *C. sativa* L. vr. *Futura 75* were hand-selected, cleaned, then subjected to hydrodistillation with a Clevenger-type apparatus (Albrigiluigi S.r.l., Stallavena, Italy) for 3 h according to the standard procedure described in the European Pharmacopoeia [108]. The essential oil was dried over anhydrous sodium sulfate to remove traces of water and then stored in dark vials at 4 °C prior to gas chromatography-mass spectrometry (GC-MS) analysis.

### 3.5. GC-FID and Gas Chromatography/Mass Spectrometry (GC/MS) Analysis

Volatile component analysis was carried out using a gas chromatography system GC 86.10 Expander (Dani) equipped with an FID detector, Rtx^®^-5 Restek capillary column (30 m × 0.25 mm i.d., 0.25 mm film thickness) (diphenyl-dimethyl polysiloxane), a split/splitless injector heated to 250 °C, and a flame ionization detector (FID) heated to 280 °C. The column temperature was maintained at 45 °C for 6 min, then programmed to increase to 250 °C at a rate of 2 °C/min and held, using an isothermal process, for 25 min; the carrier gas was He (1.0 mL/min), and 1 mL of each sample was dissolved in n-hexane (1:100 n-hexane solution) and then injected.

GC-MS analyses were performed on a Trace GC Ultra (Thermo Fisher Scientific) gas chromatography instrument equipped with an Rtx^®^-5 Restek capillary column (30 m × 0.25 mm i.d., 0.25 mm film thickness) coupled with a Polaris Q ion-trap (IT) mass spectrometry (MS) detector (Thermo Fisher Scientific, Waltham, MA). A Programmed Temperature Vaporizer (PTV) injector and a PC with a chromatography station Xcalibur (Thermo Fisher Scientific) were used. The ionization voltage was 70 eV, the source temperature was 250 °C, and the full scan acquisition in positive chemical ionization was from *m*/*z* 40 up to 400 a.m.u. at 0.43 scan s-1. The GC conditions were the same as those described above for the gas chromatography (GC-FID) analyses.

### 3.6. Identification of Essential Oil Components

The identification of the essential oil components was based on the comparison of their Kovats retention indices (RIs) and RI linear retention indices, which were determined in relation to the tR values of a homologous series of n-alkanes (C8–C40) injected under the same operating conditions as described in the literature. The MS fragmentation patterns of a single compound were taken from the NIST 02, Adams, and Wiley 275 mass spectral libraries [109] and the NIST/EPA/NIH Mass Spectral Library (NIST 05). The relative contents (%) of the components were computed as the average of the GC peak areas, which were obtained in triplicate without any corrections [110]. The identification of the cannabinoids **2**, CBN, and Δ^9^THC was based on a comparison with their t_R_ values, and MS fragmentations pattern; whenever possible, co-injection with analytical standards available in the authors’ laboratory was used. The identification of the other remaining cannabinoids was based on a comparison of their MS fragmentation patter with data from the literature [58,59].

### 3.7. Water Infusion Preparation

Dried leaves (7.0 g) were crushed and added to 100 mL of boiling distilled water in a glass beaker and left to stand at room temperature for 15 min. The mixture was then, filtered and concentrated to dryness under reduced pressure using a rotary evaporator at 40 °C to yield 88.3 mg of aqueous extract. The dry residue (WI) was submitted to Kupchan’s partitioning procedure [47] to yield four extracts: n-hexane (2.3 mg), CHCl_3_ (46.2 mg), n-BuOH (94.7 mg), and water extracts (479.7 mg), each of which was submitted to ^1^H NMR experiments. The chloroform extract was purified on a semi-preparative Nucleodur 100-5 C18 column (10 μm, 4.6 mm i.d × 250 mm) using MeOH/H_2_O (8:2) as eluent to obtain pure CBDA (1.3 mg) and CBD (4.7 mg).

### 3.8. Input File Preparation

The identification codes of proteins involved in the acute inflammatory process (GO ID: 0002526, 3789 entries) were retrieved from the Protein Data Bank. The corresponding structures were downloaded and prepared using an automated workflow previously developed by our team [66]. In detail, the unnecessary elements of each protein crystal structure (e.g., ions, solvents, and crystallization buffer components) were removed, the bond orders were fixed, and the partial charges were assigned. Then, if the original crystal structure contained a ligand, its coordinates were used to map the binding cavity around it using SiteMap [111]; otherwise, the same software was used to scan the protein surface and look up the five most probable binding sites and the highest scoring one was kept for the next steps. After the binding site was defined, the corresponding coordinates were used to build the necessary molecular docking grid, with a distance buffer of 10 Å in each direction and a spacing of 1.0 Å between the grid points.

### 3.9. Inverse Virtual Screening

Molecular docking was carried out on the target panel with AutoDock Vina software [112], with exhaustiveness of 64 and treating all open-chain bonds as active torsional bonds. At the end of the molecular docking calculations, the binding affinities were collected and normalized using a set of ten decoys. The decoy molecules shared similar chemical features with the three compounds (MW, hydrogen bond donor, and hydrogen bond acceptors) while having a different chemical structures. The normalization step, which helps to prevent false-positive results, was based on the ratio between the calculated binding affinity for the test compound (*V*_0_) and the average binding affinity value obtained when testing decoy molecules (*V_R_*); see Equation (1). This ratio generates a dimensionless parameter, called the “*V* value”, which is used to obtain a ranking of promising ligand/protein complex divisions for each investigated target that share similar chemical features with the compound of interest [63,64,65].

(1) $$V=\frac{V_{0}}{V_{R}}$$

## 4. Conclusions

This study provides useful information regarding industrial hemp leaves of the *Futura 75* cultivar, which are considered waste material, in view of their potential industrial application in food, nutraceutical, or cosmetic preparations. Via careful NMR investigations, a new 2-α-hydroxy-Δ^3,7^-cannabitriol (**1**) and seven known compounds were identified from the methanol extract.

For the cannabitriol (**1**), CBD (**2**), and CBDA (**3**), we were able to provide a possible explanation for their anti-inflammatory properties using a completely in silico approach. Inverse Virtual Screening pointed out TNFα and PPARγ as the main interactors involved in inflammatory pathways, highlighting unique binding modes for compound **1**. In addition, a possible interaction with thrombin was shown, which may explain the controversial effect of cannabinoids on blood coagulation and clot formation. The EO of *Futura 75* was characterized via GC-MS, showing a high content of CBD (28.48%) and β-caryophyllene (13.82%), the latter having anti-inflammatory activity as well.

In light of these health properties, the infusion was prepared in water, for which a high CBD content was demonstrated; this might provide dietary supplements able to aid in managing clinical symptoms related to inflammatory diseases. Moreover, the targets highlighted by our Inverse Virtual Screening experiments could disclose and support novel anti-inflammatory applications of cannabinoid derivatives. Future experiments should be aimed at clarifying the binding and the consequences of the interaction between the considered compounds (especially **1**), both with the two suggested macromolecules as well as biologically-related targets and their pathways.

## Figures and Tables

**Table 1:** ^1^H (400 MHz) and ^13^C (100 MHz) NMR data of compound **1** in CD_3_OD.

Position	δ_H_^a^	δ_C_^a^
1	3.15 t (*J* = 11.0 Hz)	48.2
2	4.73 br d (*J* = 11.0 Hz)	73.9
3	-	153.1
4	2.20 m 2.45 m	35.4
5	1.43 dd (*J* = 13.0, 3.6 Hz) 1.70 m	34.8
6	3.35 ddd (*J* = 11.0, 11.2, 3.5 Hz)	48.2
7	4.76 s 5.00 s	104.7
8	-	149.4
9	4.37 s 4.59 br s	110.6
10	1.56 s	19.1
1′	-	113.0
2′	-	157.9
3′	6.11 s	107.8
4′	-	142.9
5′	6.09 s	108.7
6′	-	157.9
1″	2.38 t (*J* = 7.7)	36.6
2″	1.55 m	32.0
3″	1.31 m	23.6
4″	1.32 m	32.7
5″	0.90 t (*J* = 6.8 Hz)	14.4

^a 1^H and ^13^C assignments aided by COSY, HSQC, and HMBC experiments.

**Table 2:** Chemical composition of essential oils (EO) hydrodistilled from the leaves of *C. sativa* L. vr. *Futura 75*.

No.	Compound	Exp. RI	Ref. RI	Area% ± DS	Abb.
1	α-Pinene	929	939	0.56 ± 0.03	BM
2	Camphene	944	954	0.02 ± 0.00	BM
3	Sabinene	972	975	0.17 ± 0.02	BM
4	Myrcene	991	990	0.11 ± 0.01	AM
5	α-Phellandrene	1000	1002	0.01 ± 0.00	MM
6	δ-3-Carene	1006	1011	0.01 ± 0.00	BM
7	α-Terpinene	1015	1017	0.02 ± 0.01	MM
8	*p*-Cymene	1023	1024	0.05 ± 0.00	MM
9	Limonene	1027	1029	0.14 ± 0.05	MM
10	1,8 Cineole	1031	1031	0.09 ± 0.01	BMO
11	*(Z)*-β-Ocimene	1041	1037	0.01 ± 0.00	AM
12	(*E*)-β-Ocimene	1052	1050	0.02 ± 0.00	AM
13	γ-Terpinene	1060	1059	0.02 ± 0.00	MM
14	Terpinolene	1087	1088	0.02 ± 0.01	MM
15	*p*-Cymenene	1089	1091	0.02 ± 0.00	MM
16	Linalool	1101	1096	0.10 ± 0.01	AM
17	Nonanal	1106	1100	0.02 ± 0.00	OT
18	endo-Fenchol	1112	1116	0.02 ± 0.00	BMO
19	*trans*-Pinene-hydrate	1121	1122	0.01 ± 0.00	BMO
20	*trans*-Pinocarveol	1138	1139	0.03 ± 0.00	BMO
21	Camphor	1144	1146	0.01 ± 0.00	BMO
22	Borneol	1166	1169	0.06 ± 0.00	BMO
23	Terpinen-4-ol	1177	1177	0.08 ± 0.00	BMO
24	p-Cymen-8-ol	1187	1182	0.01 ± 0.01	MMO
25	α-Terpineol	1190	1188	0.03 ± 0.00	MMO
26	Myrtenol	1195	1195	0.02 ± 0.00	BMO
27	Estragole (Methyl chavicol)	1198	1195	0.01 ± 0.01	OT
28	*trans*-Pulegol	1218	1214	0.06 ± 0.01	MMO
29	*trans*-Carveol	1220	1216	0.01 ± 0.00	MMO
30	Linalool acetate	1260	1257	0.04 ± 0.00	AMO
31	Eugenol	1359	1359	0.04 ± 0.00	MMO
32	α-Ylangene	1369	1375	0.12 ± 0.01	BS
33	α-Copaene	1373	1376	0.09 ± 0.02	BS
34	β-Elemene	1388	1390	0.11 ± 0.01	MS
35	β-Longipinene	1402	1400	0.55 ± 0.04	BS
36	*Z*-Caryophyllene	1407	1408	0.13 ± 0.03	BS
37	β-Caryophyllene	1419	1419	13.82 ± 0.47	BS
38	α-*trans*-Bergamotene	1436	1434	1.58 ± 0.04	MS
39	α-Humulene	1453	1454	5.33 ± 0.17	MS
40	allo-Aromadendrene	1459	1460	1.46 ± 0.10	BS
41	dehydro-Aromadendrene	1460	1462	0.45 ± 0.02	BS
42	γ-Himachalene	1483	1482	3.5 ± 0.14	BS
43	α-Selinene	1492	1498	1.97 ± 0.03	BS
44	β-Himacalene	1508	1500	0.22 ± 0.03	BS
45	δ-Amorphene	1512	1512	0.73 ± 0.06	BS
46	γ-Cadinene	1516	1513	0.32 ± 0.06	BS
47	δ-Cadinene	1523	1523	0.32 ± 0.03	BS
48	*trans*-Cadina-1,4-diene	1532	1534	0.92 ± 0.03	BS
49	α-Cadinene	1539	1538	1.22 ± 0.08	BS
50	α-Calacorene	1541	1545	0.1 ± 0.01	BS
51	Selina-3,7(11)-diene	1543	1546	0.04 ± 0.00	BS
52	Italicene epoxide	1549	1548	1.0 ± 0.01	BSO
53	(*E*)-Nerolidol	1567	1563	1.01 ± 0.04	ASO
54	Caryophyllene oxide	1581	1583	5.7 ± 0.39	BSO
55	Spathulenol	1583	1578	0.09 ± 0.01	BSO
56	Viridiflorol	1595	1592	0.55 ± 0.06	BSO
57	Ledol	1599	1602	0.45 ± 0.09	BSO
58	Humulene epoxide II	1606	1608	1.7 ± 0.16	MSO
59	Isolongifolan-7-α-ol	1616	1619	0.83 ± 0.02	BSO
60	allo-Aromadendrene-epoxide	1634	1641	4.41 ± 0.37	BSO
61	Caryophylla-4(12),8(13)-dien-5α-ol	1638	1640	2.43 ± 0.18	BSO
62	Selina-3,11-dien-6-α-ol	1643	1644	0.63 ± 0.07	BSO
63	Desmethoxy encecalin	1651	1647	0.94 ± 0.11	OT
64	α-Bisabolol oxide B	1661	1658	5.12 ± 0.33	BSO
65	(*Z*)-Caryophyllene-14-hydroxy	1674	1667	3.44 ± 0.10	BSO
66	epi-α-Bisabolol-	1684	1684	0.3 ± 0.04	MSO
67	Eudesm-7(11)-en-4-ol	1693	1700	0.23 ± 0.02	BSO
68	(*2E*,*6E*)-Farnesyl acetate	1847	1846	0.12 ± 0.03	OT
69	(*5E*,*9E*)-Farnesyl acetone	1919	1913	0.15 ± 0.02	OT
70	*trans*-Phytol	2113	2104	0.22 ± 0.05	OT
71	Linoleic acid	2137	2133	0.12 ± 0.01	OT
72	Cannabidivarin	2217		0.72 ± 0.08	CB
73	Cannabicitran	2271		1.56 ± 0.1	CB
74	U (314, 299, 271, 258, 243, **231**, 174)	2332		0.2 ± 0.01	U
75	Cannabiclyclol	2367		0.26 ± 0.02	CB
76	Cannabidiol (CBD)	2432	2430	28.48 ± 3.02	CB
77	Cannabichromene	2437		0.61 ± 0.12	CB
78	Dronabinol (Δ^8^-THC)	2484		0.11 ± 0.01	CB
79	U (330, 312, 247, **205**, 148, 135)	2488		0.13 ± 0.01	U
80	Δ-THC	2521		0.15 ± 0.03	CB
81	Cannabigerol	2587		0.08 ± 0.01	CB
82	Cannabinol	2587		0.14 ± 0.03	CB
83	Heptacosane	2699	2700	0.2 ± 0.00	OT
84	Nonacosane	2899	2900	1.04 ± 0.03	OT
	Total identified (%)			97.45	
	Oil yield (%)			0.1	
	Monoterpene Hydrocarbons			1.28	
	Oxigenate monoterpenes			0.52	
	Sesquiterpene Hydrocarbons			32.71	
	Oxigenate sesquiterpenes			28.04	
	Cannabinoids			32.11	
	Others			2.79	

Abbreviations: **AM**—aliphatic monoterpenes; **MM**—monocyclic monoterpenes; **BM**—bi- and tricyclic monoterpenes; **AMO**—aliphatic monoterpenoids; **MMO**—monocyclic monoterpenoids; **BMO**—bi-and tricyclic monoterpenoids; **AS**—aliphatic sesquiterpenes; **MS**—monocyclic sesquiterpenes; **BS**—bi- and tricyclic sesquiterpenes; **ASO**—aliphatic sesquiterpenoids; **MSO**—monocyclic sesquiterpenoids; **BSO**—bi- and tricyclic sesquiterpenoids, **CB**—cannabinoids, **OT**—others. **SD**—standard deviation; **Exp. RI**—experimental retention index; **Ref.** **RI**—literature data, **U**—unknown (base peak in bold).

**Table 3:** Top-scoring target for each compound and the corresponding binding affinity.

Compound	Top-Scored Target	PDB	Binding Affinity
**1**	Tumor necrosis factor	6X83 [67]	−9.2
**2**	Tumor necrosis factor	7KPA [68]	−9.7
**3**	Tumor necrosis factor	7KPA [68]	−10.1

**Table 4:** The most retrieved targets shared between **1**, **2**, and **3** and the corresponding best binding affinities.

UniProt ID	Molecule Name	Binding Affinity (kcal/mol) (PDB ID)		
		1	2	3
P00734	Prothrombin/Thrombin	−8.4 (6ZUX [69])	−8.6 (6ZV8 [69])	−8.5 (1RD3 [70])
P37231	Peroxisome proliferator-activated receptor gamma	−8.6 (2ZK6 [71])	−9.2 (4PRG [72])	−9.4 (4PRG [72])

## Data Availability

Not applicable.

## Supplementary Materials

The following supporting information can be downloaded at: https://www.mdpi.com/article/10.3390/plants11131671/s1, Figure S1: ^1^H NMR (CD_3_OD, 400 MHz) of 2α-hydroxy-Δ^3,7^-cannabitriol (**1**); Figure S2: ^13^C NMR (CD_3_OD, 100 MHz) of (**1**)*;* Figure S3: COSY spectrum (CD_3_OD, 400 MHz) of (**1**)*;* Figure S4: HSQC spectrum (CD_3_OD, 400 MHz) of (**1**); Figure S5. HMBC spectrum (CD_3_OD, 400 MHz) of (**1**); Figure S6: ^1^H NMR (CD_3_OD, 400 MHz) of Cannabidiol (**2**); Figure S7: ^1^H NMR (CD_3_OD, 400 MHz) of Cannabidiolic acid (**3**); Figure S8: ^1^H NMR (C_5_D_5_N, 500 MHz) of α-Cannabispiranol (**4**); Figure S9: ^1^H NMR (C_5_D_5_N, 500 MHz) of β-Cannabispiranol (**5**); Figure S10: ^1^H NMR (CD_3_OD, 500 MHz) of Canniprene (**6**); Figure S11: ^1^H NMR (CD_3_OD, 400 MHz) of Cannabiripsol (**7)**; Figure S12: ^1^H NMR (CD_3_OD, 400 MHz) of Cannflavine B (**8**); Figure S13: GC-MS–Total ion Essential oil; Figure S14–S16: Superposition of **1**, **2**, and **3** with the co-crystallized ligands in the PDB structure (if available).
