# Supplementary material for: Phytochemical Analysis of the Methanolic Extract and Essential Oil from Leaves of Industrial Hemp Futura 75 Cultivar: Isolation of a New Cannabinoid Derivative and Biological Profile Using Computational Approaches
Source: Plants (Basel). 2022 Jun 24;11(13):1671. doi: 10.3390/plants11131671 (PMC9269227; doi:10.3390/plants11131671)
Supplement: Supplementary file 1 [file plants-11-01671-s001.zip › plants-1779137-supplementary.pdf]

# Phytochemical analysis of methanol extracts and of essential oil from leaves of industrial hemp *Futura 75* cultivar: Isolation of a new cannabinoid derivative and biological profile using computational approaches.

**Simona De Vita[1,#], Claudia Finamore[2,#], Maria Giovanna Chini[3], Gabriella Saviano[3], Vincenzo De Felice[3], Simona De Marino[2], Gianluigi Lauro[1], Agostino Casapullo[1], Francesca Fantasma[3], Federico Trombetta[4], Giuseppe Bifulco[1*], Maria Iorizzi[3*]**

[1] Department of Pharmacy, University of Salerno, Via Giovanni Paolo II 132, 84084, Fisciano, Salerno, Italy; sdevita@unisa.it (S.D.V.); glauro@unisa.it (G.L.); casapullo@unisa.it (A.C.)

[2] Department of Pharmacy, University of Naples, Via Domenico Montesano, 49, 80131 Naples, Italy; claudia.finamore@unina.it (C.F.); sidemari@unina.it (S.D.M.)

[3] Department of Biosciences and Territory, University of Molise, Contrada Fonte Lappone, Pesche, 86090 Isernia, Italy; mariagiovanna.chini@unimol.it (M.G.C.); saviano@unimol.it (G.S.); defelice@unimol.it (V.D.F.); fantasma@unimol.it (F.F.)

[4] Società Cooperativa Agricola MarcheSana, Località San Biagio 40, 61032, Fano, Pesaro-Urbino, Italy

[#] These authors contributed equally to this work

* Correspondence: bifulco@unisa.it (G.B.); Tel.: (+39)089969741 and iorizzi@unimol.it (M.I.); Tel.: (+39)0874404100.

## $^1$H NMR ($CD_3OD$, 400 MHz) of 2$\alpha$-hydroxy-$\Delta^{3,7}$-cannabitriol (1)

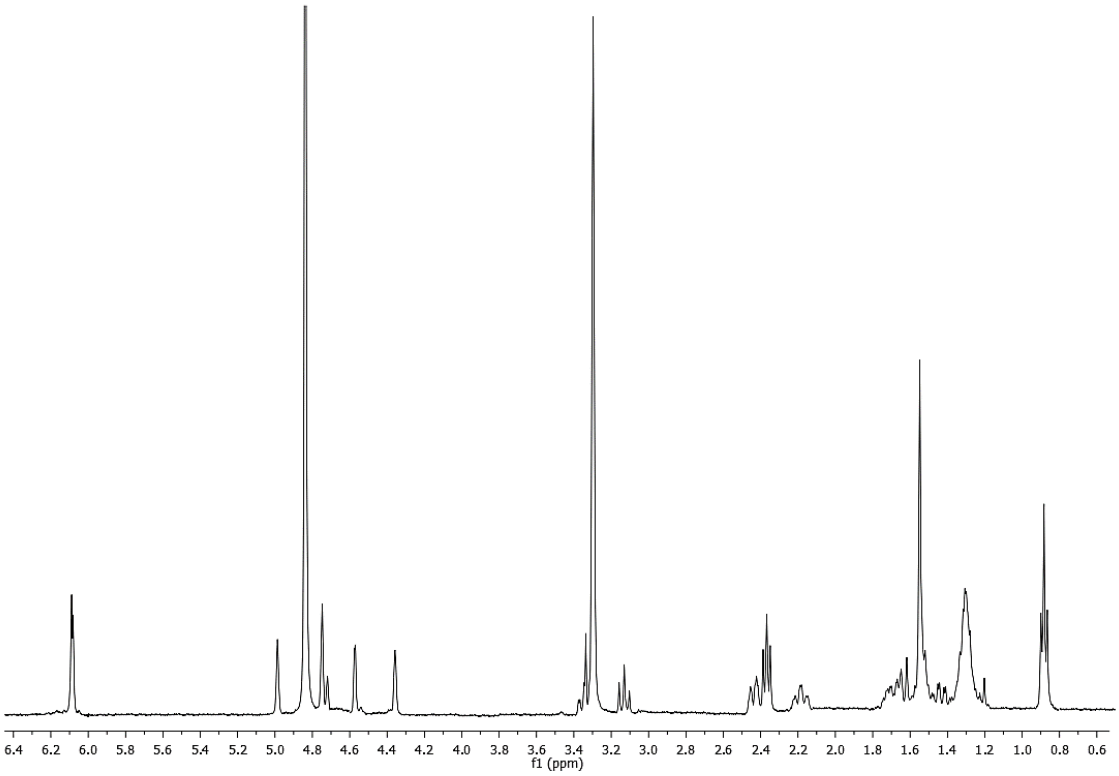


Figure S1. $^1$H NMR ($CD_3OD$, 400 MHz) of 2$\alpha$-hydroxy-$\Delta^{3,7}$-cannabitriol (**1**).

## $^{13}$C NMR (CD$_3$OD, 100 MHz) of 2$\alpha$-hydroxy-$\Delta^{3,7}$-cannabitriol (1)

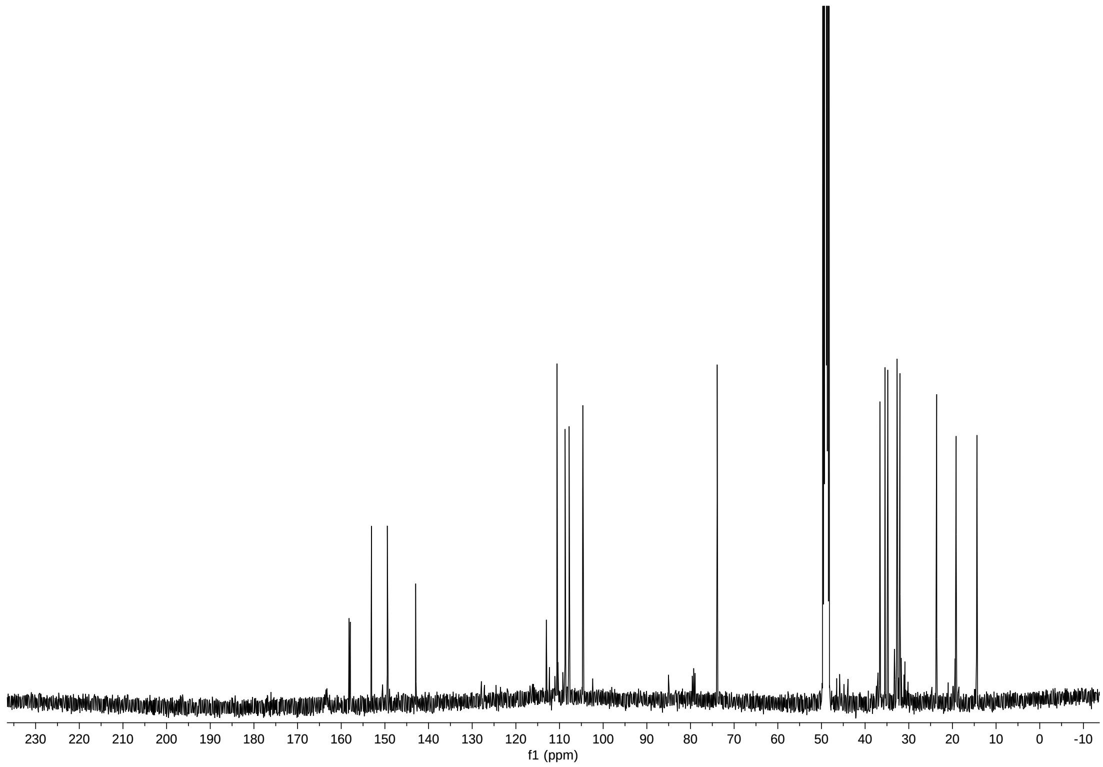


Figure S2. $^{13}$C NMR (CD$_3$OD, 100 MHz) of 2$\alpha$-hydroxy-$\Delta^{3,7}$-cannabitriol (**1**).

## COSY spectrum (CD$_3$OD, 400 MHz) of 2$\alpha$-hydroxy-$\Delta^{3,7}$-cannabitriol (1)

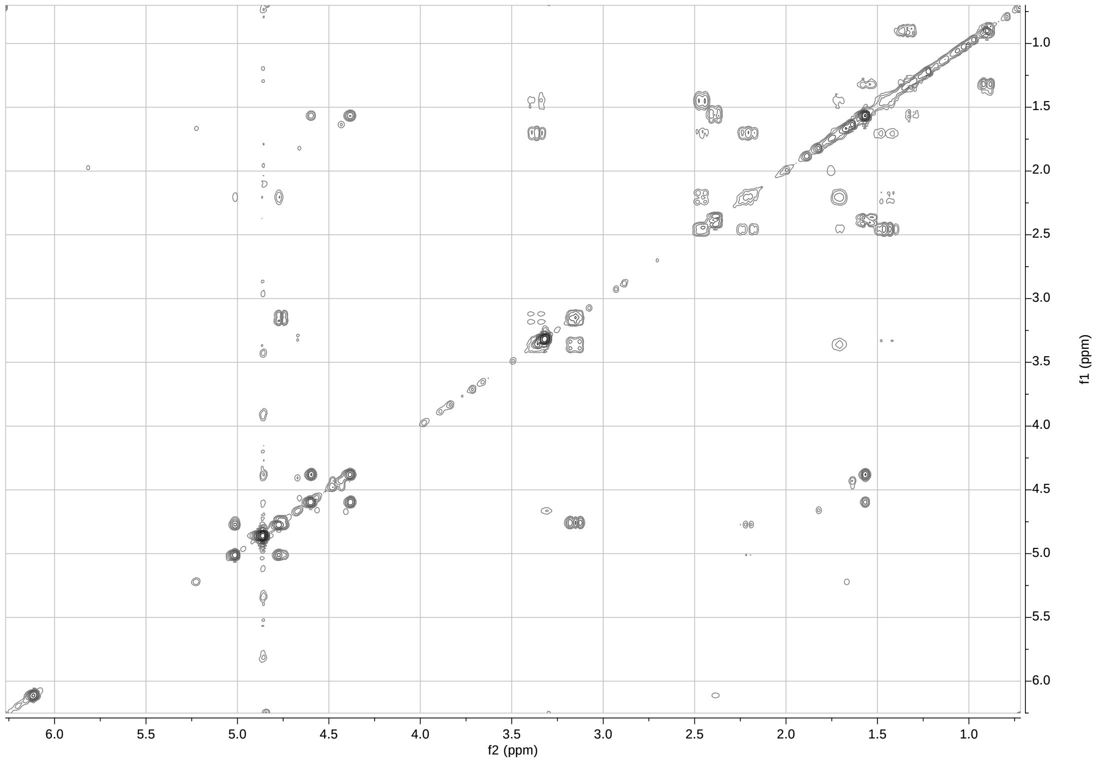


Figure S3. COSY spectrum (CD$_3$OD, 400 MHz) of 2$\alpha$-hydroxy-$\Delta^{3,7}$-cannabitriol (**1**).

## HSQC spectrum ($CD_3OD$, 400 MHz) of 2$\alpha$-hydroxy-$\Delta^{3,7}$-cannabitriol (**1**)

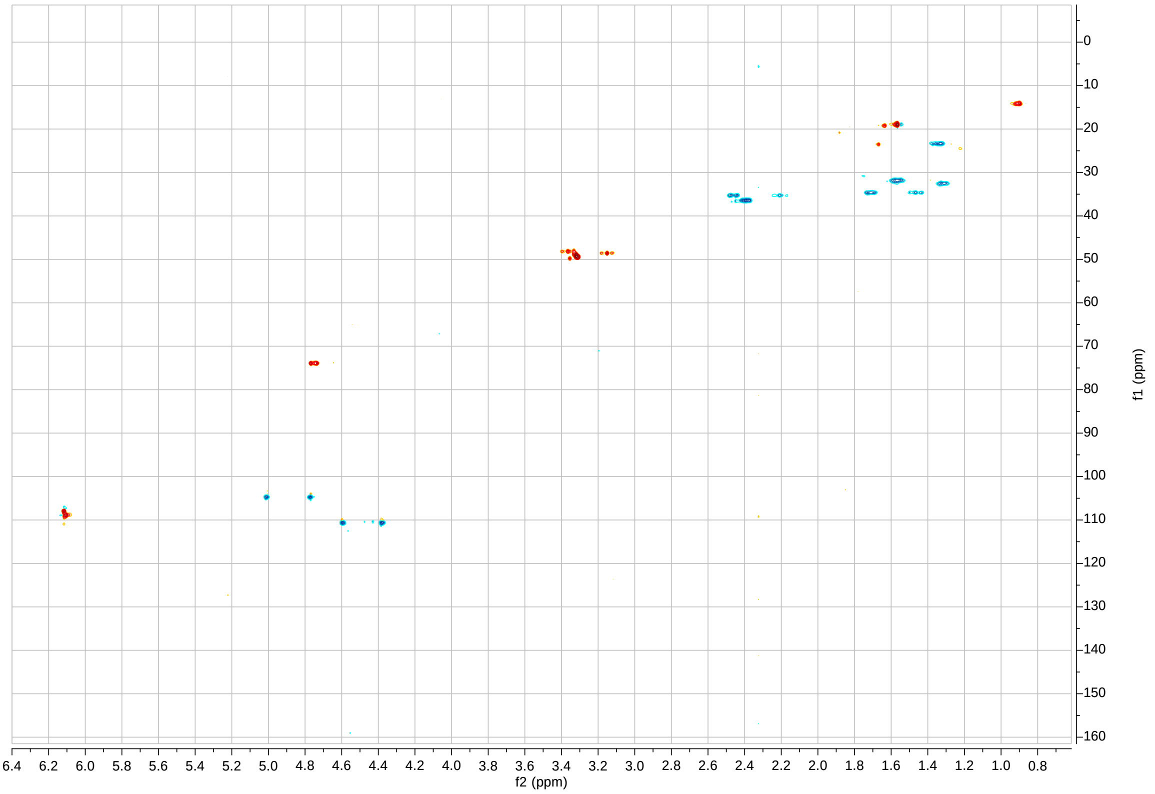


Figure S4. HSQC spectrum ($CD_3OD$, 400 MHz) of 2$\alpha$-hydroxy-$\Delta^{3,7}$-cannabitriol (**1**).

## HMBC spectrum ($CD_3OD$, 400 MHz) of 2$\alpha$-hydroxy-$\Delta^{3,7}$-cannabitriol (**1**)

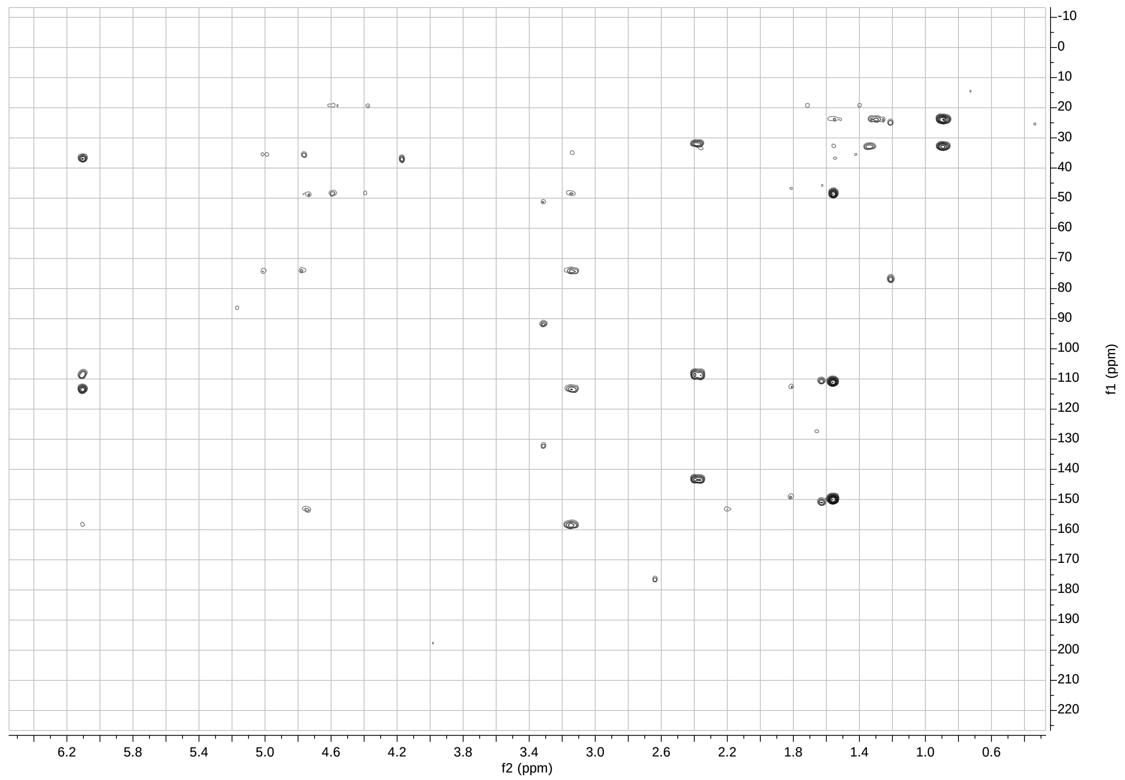


Figure S5. HMBC spectrum ($CD_3OD$, 400 MHz) of 2$\alpha$-hydroxy-$\Delta^{3,7}$-cannabitriol (**1**).

## $^1$H NMR ($CD_3OD$, 400 MHz) of Cannabidiol (2)

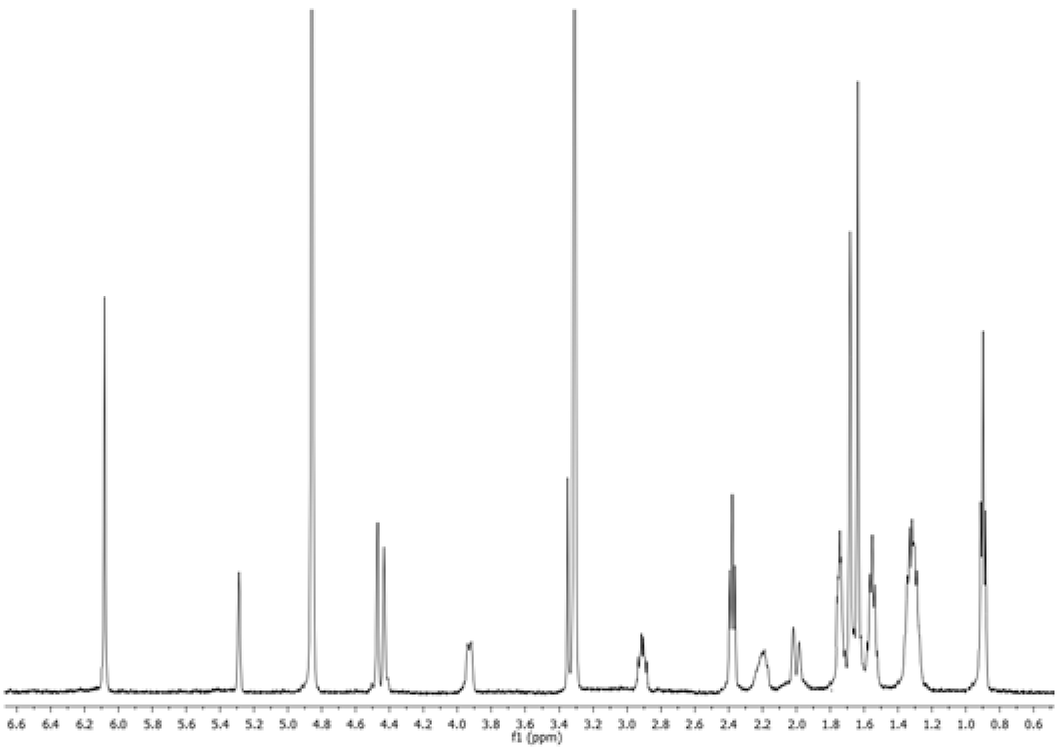


Figure S6. $^1$H NMR ($CD_3OD$, 400 MHz) of Cannabidiol (**2**).

## $^1$H NMR ($CD_3OD$, 400 MHz) of Cannabidiolic acid (3)

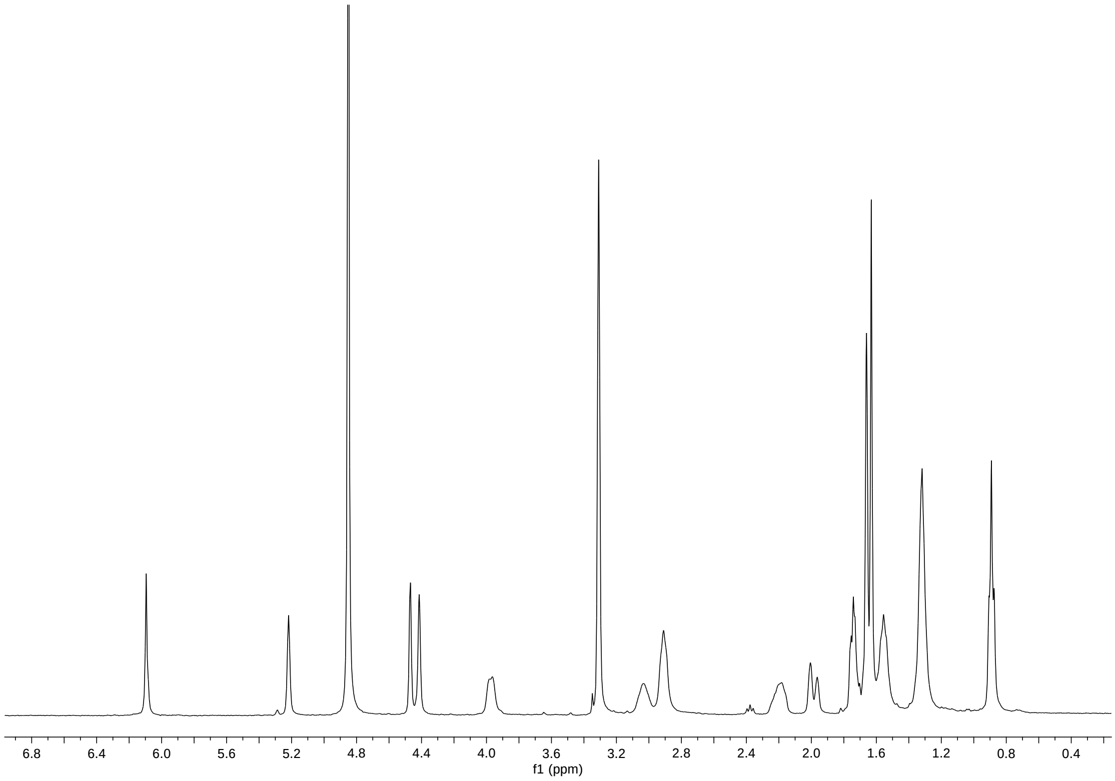


Figure S7. $^1$H NMR ($CD_3OD$, 400 MHz) of Cannabidiolic acid (**3**).

**$^1$H NMR ($C_5D_5N$, 500 MHz) of α-Cannabispiranol (4)**

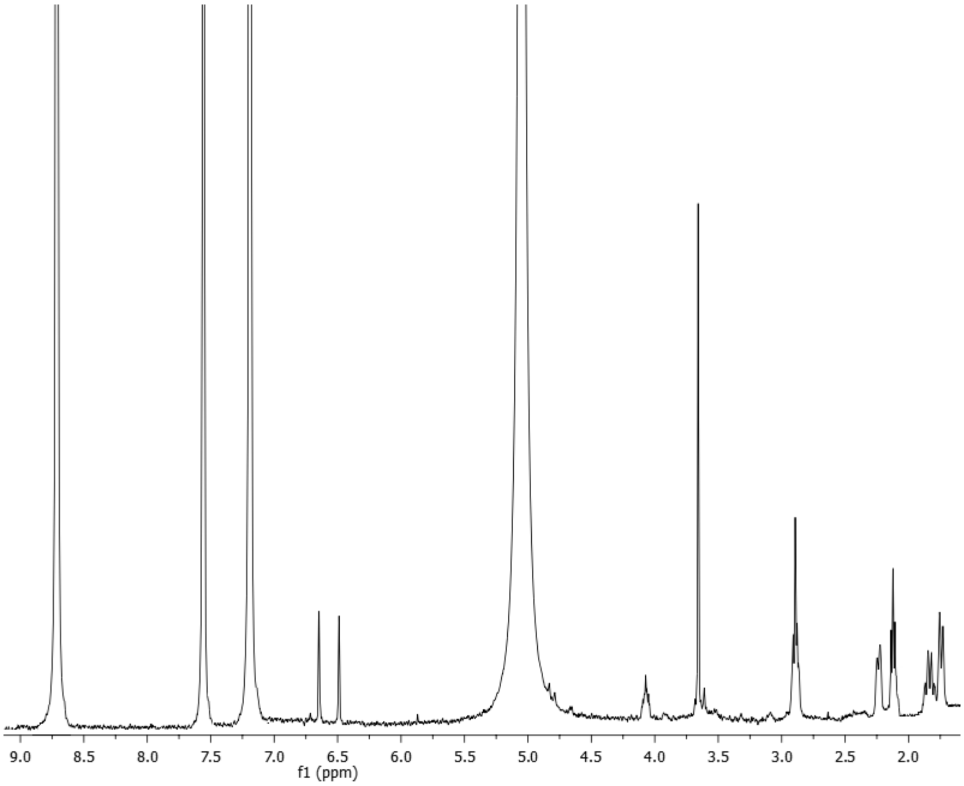


Figure S8. $^1$H NMR ($C_5D_5N$, 500 MHz) of α-Cannabispiranol (**4**).

**$^1$H NMR ($C_5D_5N$, 500 MHz) of β-Cannabispiranol (5)**

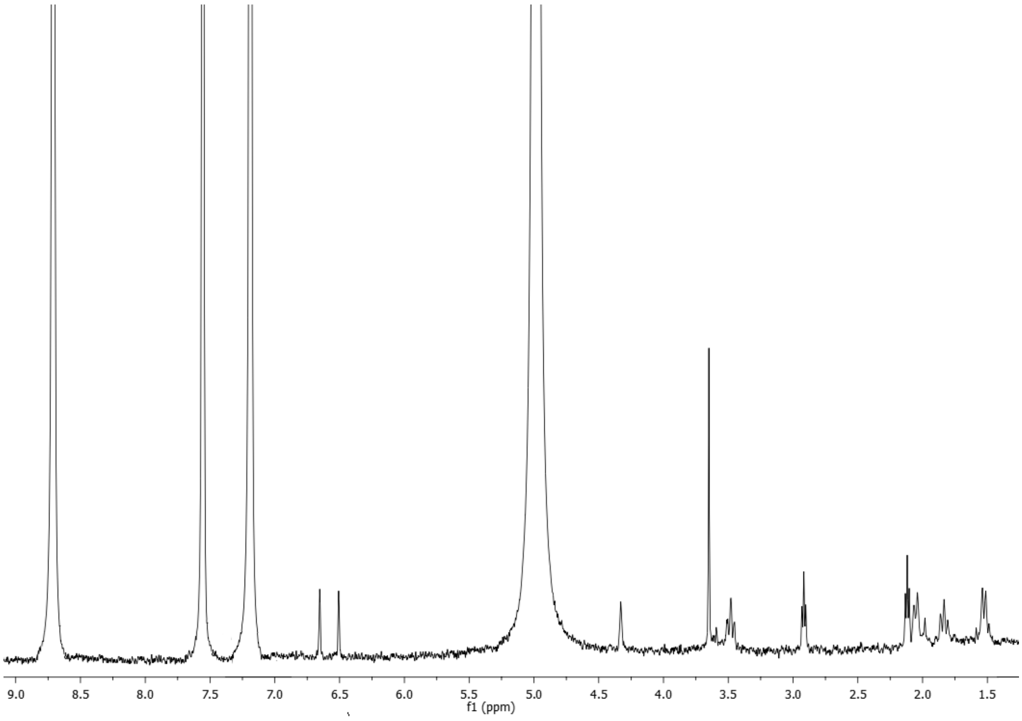


Figure S9. $^1$H NMR ($C_5D_5N$, 500 MHz) of β-Cannabispiranol (**5**).

## $^{1}H$ NMR ($CD_3OD$, 500 MHz) of Canniprene (6)

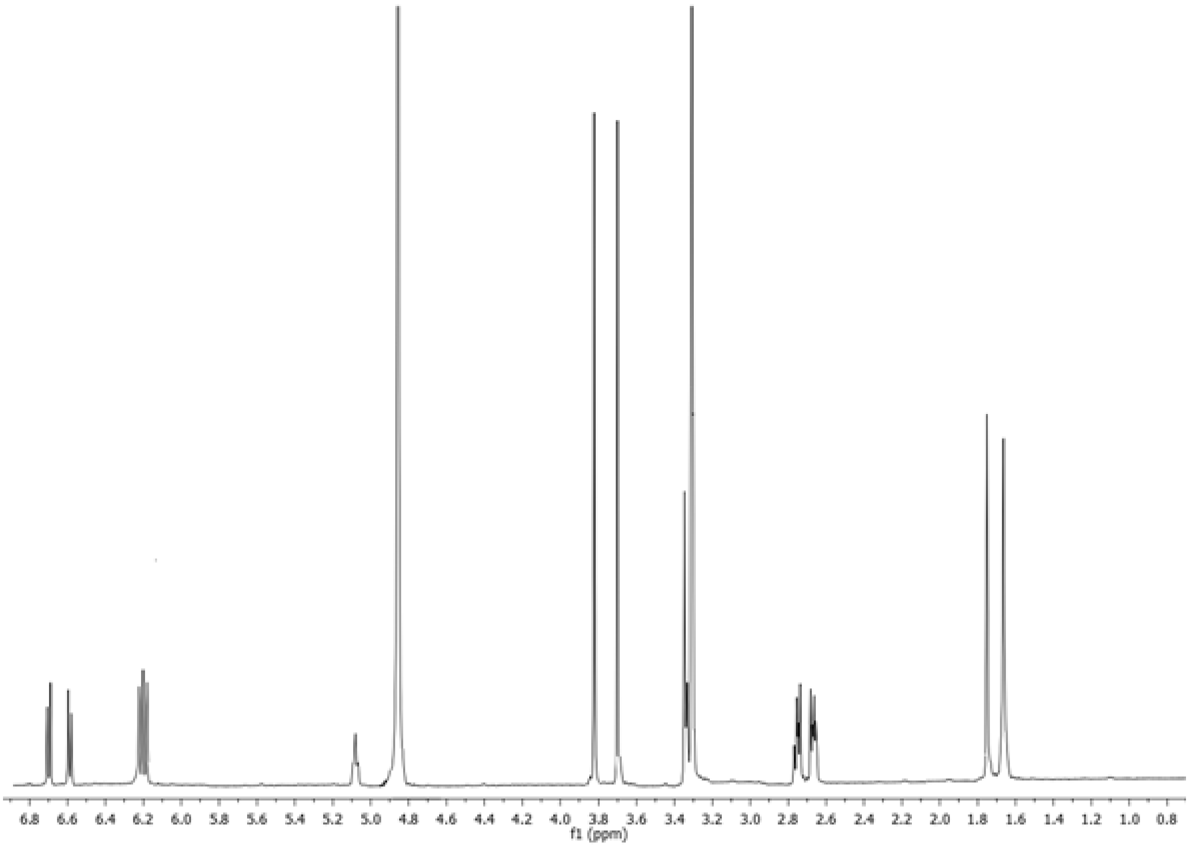


Figure S10. $^{1}H$ NMR ($CD_3OD$, 500 MHz) of Canniprene (**6**).

## $^{1}H$ NMR ($CD_3OD$, 400 MHz) of Cannabiripsol (7)

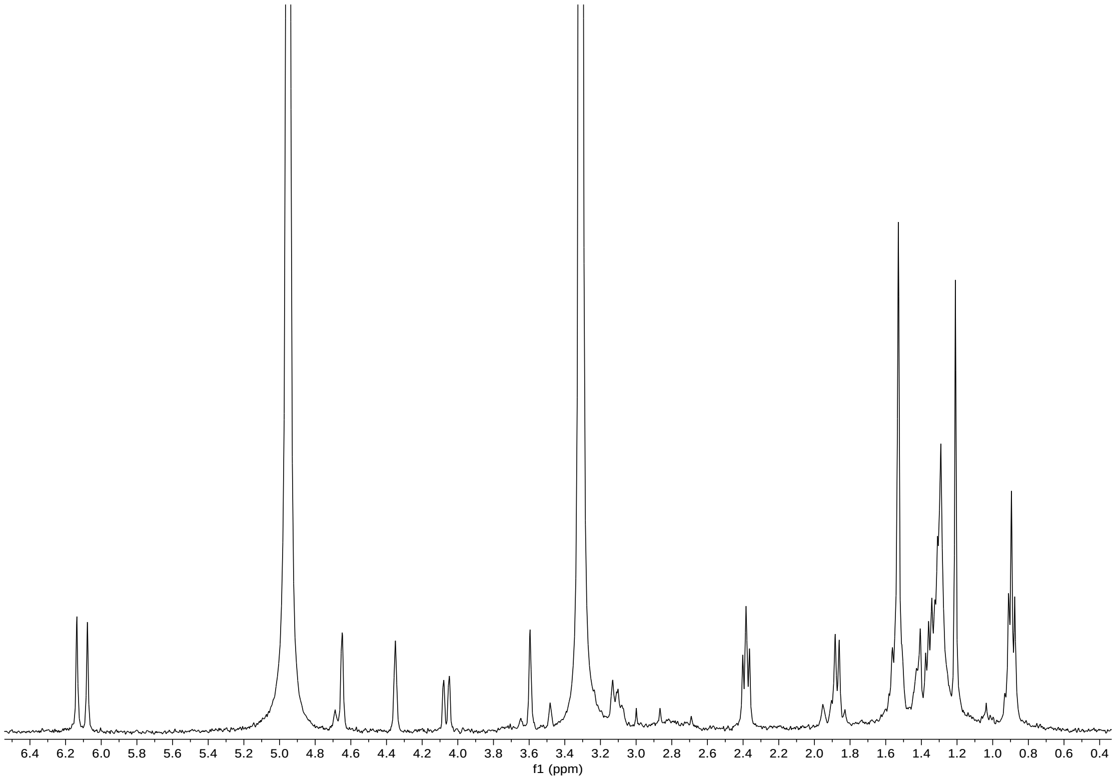


Figure S11. $^{1}H$ NMR ($CD_3OD$, 400 MHz) of Cannabiripsol (**7**).

**$^1$H NMR ($CD_3OD$, 400 MHz) of Cannflavine B (8)**

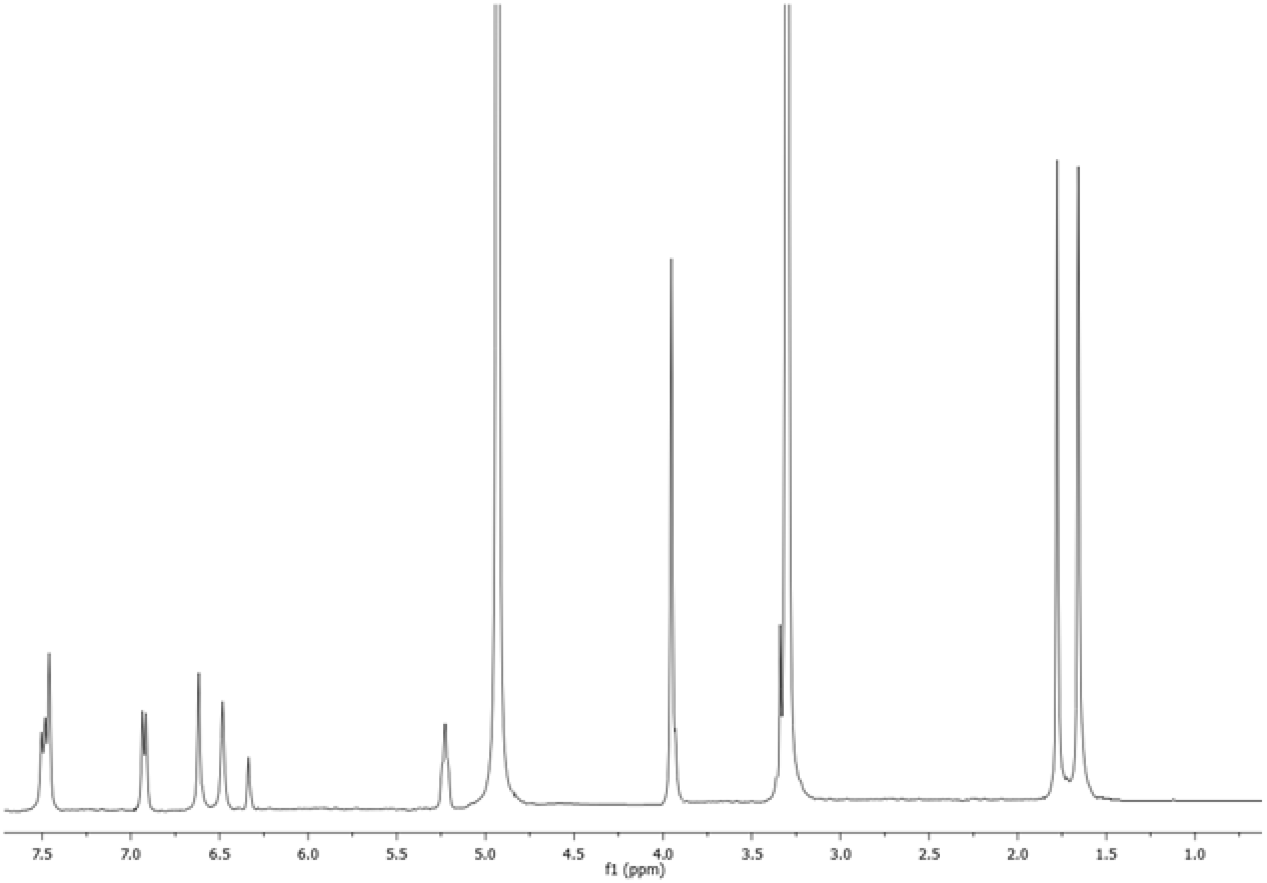


Figure S12. $^1$H NMR ($CD_3OD$, 400 MHz) of Cannflavine B (**8**).

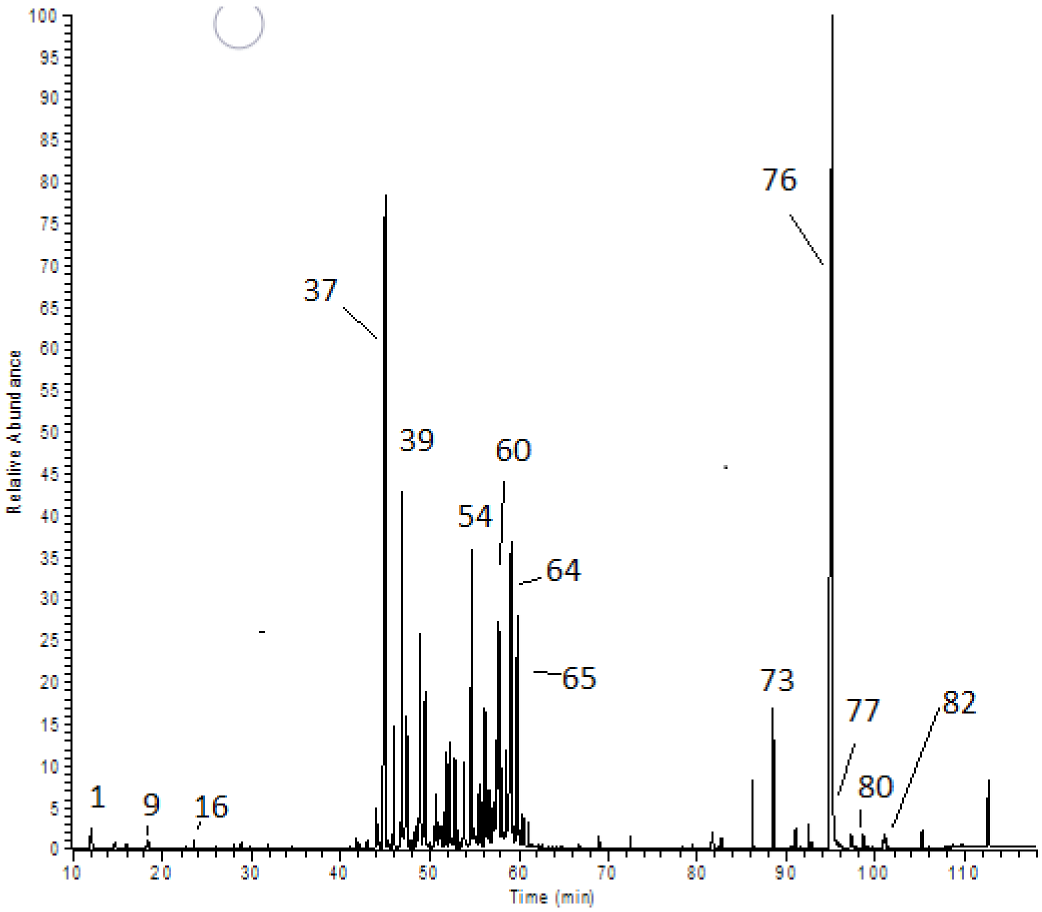


Figure S13. GC-MS TIC chromatogram of *C. sativa* L. cv. *Futura 75*. Numbers refer to those reported in Table 2.

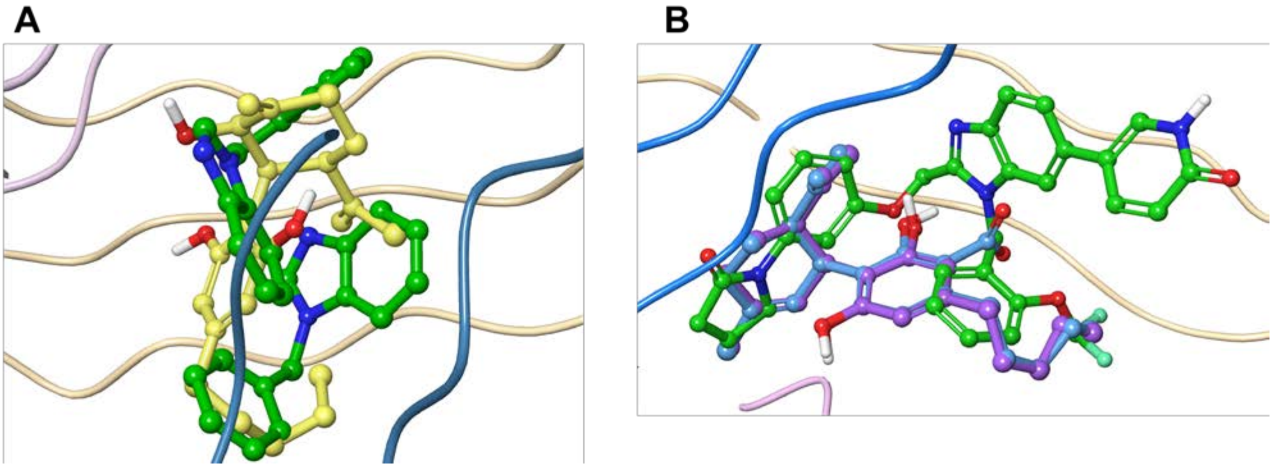


Figure S14. Superimposition of **1** (**A**) and **2** and **3** (**B**) with the co-crystallized inhibitors contained in 6X83 and 7KPA, respectively.

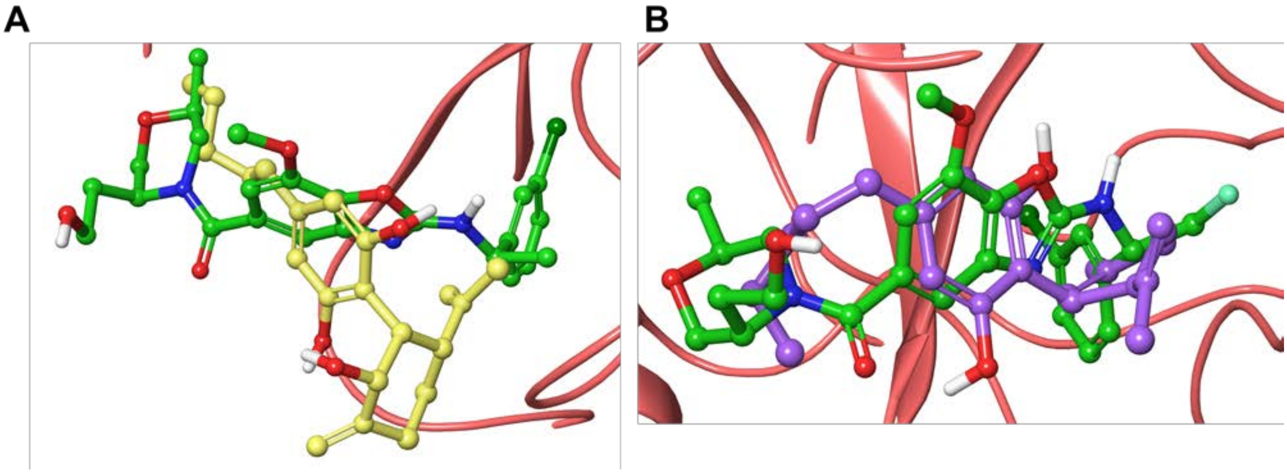


Figure S15. Superimposition of **1** (**A**) and **2** with the co-crystallized inhibitors contained in 6ZUX and 6ZV8, respectively.

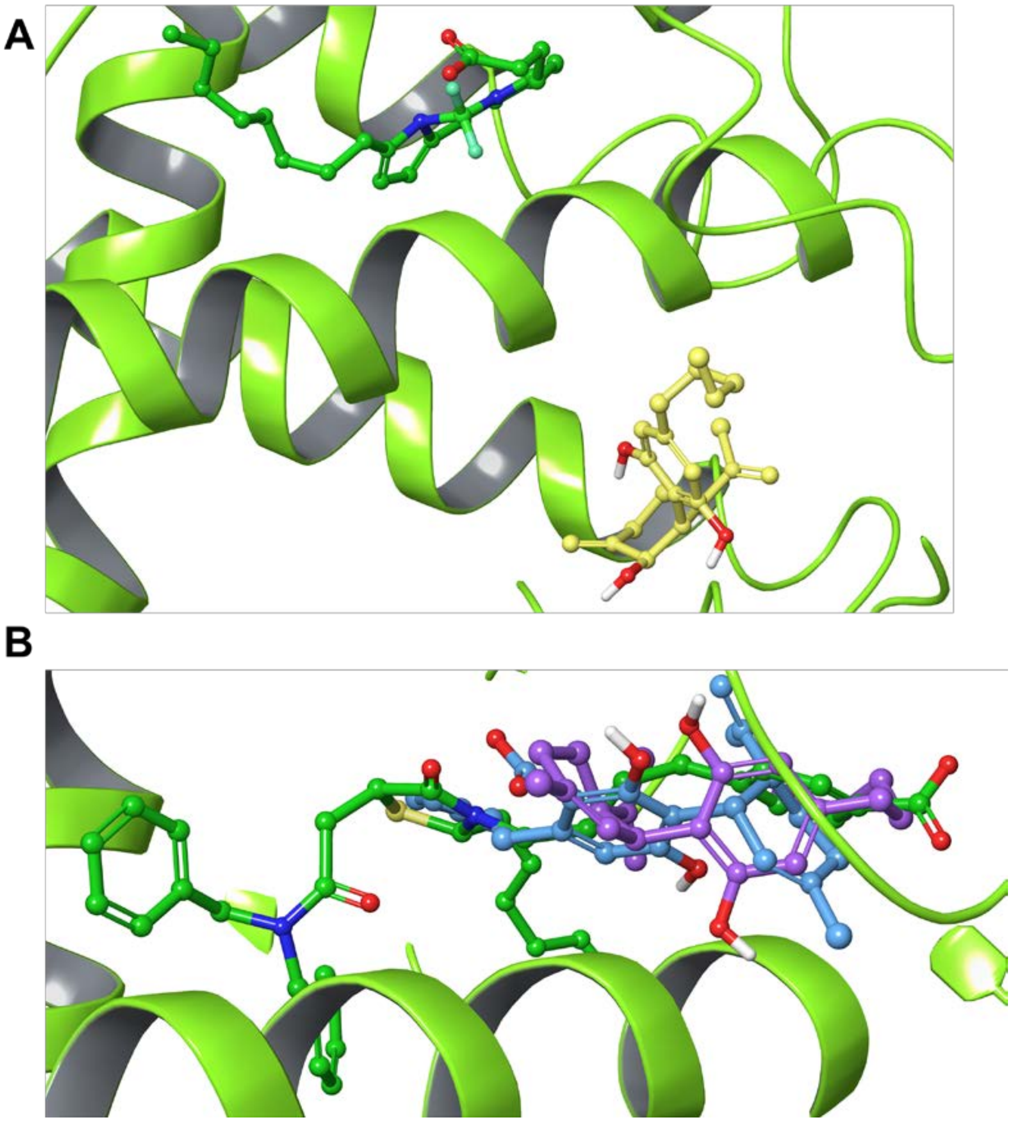


Figure S16. Superimposition of **1** (**A**) and **2** and **3** (**B**) with the co-crystallized inhibitors contained in 2ZK6 and 4PRG, respectively.
